# Predicting RTS,S Vaccine-Mediated Protection from Transcriptomes in a Malaria-Challenge Clinical Trial

**DOI:** 10.3389/fimmu.2017.00557

**Published:** 2017-05-23

**Authors:** Robert A. van den Berg, Margherita Coccia, W. Ripley Ballou, Kent E. Kester, Christian F. Ockenhouse, Johan Vekemans, Erik Jongert, Arnaud M. Didierlaurent, Robbert G. van der Most

**Affiliations:** ^1^GSK Vaccines, Rue de l’Institut, Rixensart, Belgium; ^2^Walter Reed Army Institute of Research, Silver Spring, MD, USA

**Keywords:** malaria, systems biology, transcriptome, vaccine, clinical trial, interferon gamma, RTS,S, adjuvant

## Abstract

The RTS,S candidate malaria vaccine can protect against controlled human malaria infection (CHMI), but how protection is achieved remains unclear. Here, we have analyzed longitudinal peripheral blood transcriptome and immunogenicity data from a clinical efficacy trial in which healthy adults received three RTS,S doses 4 weeks apart followed by CHMI 2 weeks later. Multiway partial least squares discriminant analysis (N-PLS-DA) of transcriptome data identified 110 genes that could be used in predictive models of protection. Among the 110 genes, 42 had known immune-related functions, including 29 that were related to the NF-κB-signaling pathway and 14 to the IFN-γ-signaling pathway. Post-dose 3 serum IFN-γ concentrations were also correlated with protection; and N-PLS-DA of IFN-γ-signaling pathway transcriptome data selected almost all (44/45) of the representative genes for predictive models of protection. Hence, the identification of the NF-κB and IFN-γ pathways provides further insight into how vaccine-mediated protection may be achieved.

## Introduction

The medical burden of malaria disease remains high, most notably in Africa, where most of the deaths due to malaria occur in children under 5 years of age (1). Malaria infection is initiated by the mosquito bite, from which *Plasmodium* sporozoites pass to the liver *via* the blood to infect hepatocytes. The entry into hepatocytes is mediated by circumsporozoite protein (CSP); a protein that is highly expressed at the surface of the sporozoite (2). CSP is also the target of the RTS,S candidate malaria vaccine, in which RTS,S is a recombinant antigen derived from CSP from *Plasmodium falciparum* and the hepatitis B surface antigen. The selection of CSP was informed by the results from vaccination with inactivated sporozoites (3). Vaccination with inactivated sporozoites can result in sterile immunity, which has been associated with activation of CSP-specific cell-mediated immunity (CMI) and production of CSP-specific antibodies (4–7).

RTS,S has been shown to provide partial protection against clinical and severe disease to infants and young children (8) in a phase III field trial. Protective efficacy has also been demonstrated in adults in phase II trials after controlled human malaria infection (CHMI; by bites received from *Plasmodium*-infected mosquitoes) and in the field (8). The degree of protection provided by RTS,S is dependent on the type of vaccine adjuvant in its composition (9, 10): Adjuvant System AS01 is currently selected for the RTS,S composition and has replaced AS02 (11). Both AS01 and AS02 contain the immunostimulants MPL, a toll-like receptor 4 agonist and the saponin QS-21. The two Adjuvant Systems differ in that AS01 is formulated with liposomes and AS02 is formulated as an oil-in-water emulsion (11). In AS01, the combination of MPL and QS-21 enhances antibody and T-cell responses to vaccine antigens, potentially *via* the transient stimulation of the innate immune system which induces efficient antigen-presenting dendritic cells (12, 13).

A recent analysis of the RTS,S phase III trial has identified the CSP-specific serum antibody concentration as a surrogate of RTS,S-mediated protection (14). Some, but not all, RTS,S studies suggest that CSP-specific CMI measured in peripheral blood is also associated with protection (9, 10, 15–19). In some RTS,S studies, CSP-specific interferon (IFN) gamma (IFN-γ) induction (detected by ELISPOT in cultures of PBMCs) has been associated with protection against malaria-related endpoints both in the field and in the CHMI setting (10, 20, 21). IFN-γ-ELISPOT responses to vaccination also appear to be enhanced when RTS,S is combined with AS01 or AS02 (10, 20, 22). CSP-specific CD4^+^ T cells have also been associated with protection in some CHMI and field trials (10, 18, 23, 24). However, those associations have been made with CD4^+^ T cells that predominantly express IL-2 or TNF-α, rather than IFN-γ. Therefore, questions remain as to whether there are other molecular markers in blood samples that can better predict and perhaps explain RTS,S-mediated protection.

**Figure 1:**
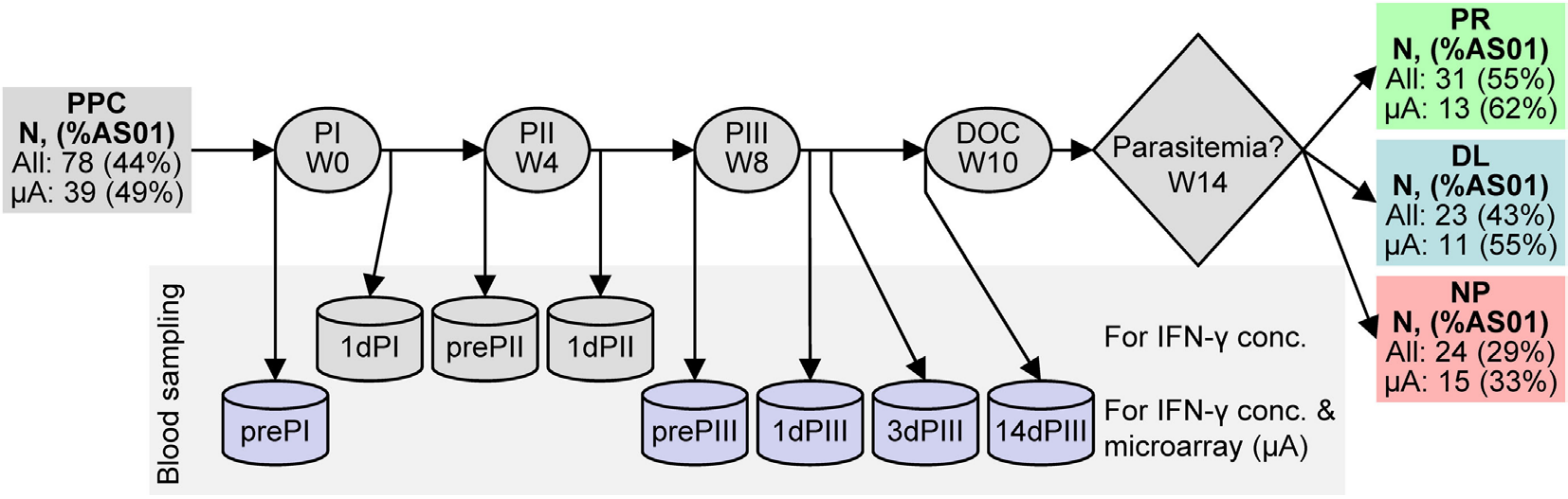
**Schematic representation of RTS,S candidate vaccine clinical trial design and efficacy results**. RTS,S vaccine injections were performed at weeks (W) 0, 4, and 8. Blood was sampled (represented by *can* shapes) before the first, second, and third vaccine injections (prePI, prePII, and prePIII, respectively), one day after the first, second, and third vaccine injections (1dPI, 1dPII, and 1dPIII, respectively), and 3 and 14 days after the third vaccine injection (3dPIII, 10dPIII, and 14dPIII, respectively). Controlled human malaria infection (CHMI) was performed at 14dPIII (i.e., week 10; day of challenge, DOC) and the onset of parasitemia was followed up to week 14. Serum IFN-γ concentrations were measured at all of the time points, whereas RNA expression was evaluated at prePI, prePIII, 1dPIII, 3dPIII, and 14dPIII (purple cans). The numbers of subjects in the per-protocol cohort (PPC) at study entry and after the outcome of CHMI [either protected (PR), non-protected (NP), or delayed onset of parasitemia (DL)] are indicated (*all* subjects) and correspond to those subjects who also provided blood samples for IFN-γ measurements. The numbers of subjects from which transcriptome data from microarrays (μA subjects) were derived are indicated below *all* subjects. The percentages of RTS,S/AS01 recipients over recipients of either vaccine (%AS01) are indicated in parentheses for *all* subjects and μA subjects after vaccination and after challenge. Note that all 12 non-vaccinated control subjects developed parasitemia within the follow-up period after CHMI (not shown).

Systems biology approaches can interrogate information from large data sets so as to identify predictive signatures of outcomes such as protection, or immunological correlates of protection (25–28). In the present study, multiway partial least squares discriminant analysis (N-PLS-DA) (29–31) was selected to identify relationships between transcriptome data and protection against parasitemia in clinical-trial recipients of RTS,S (10). In that trial, 78 malaria-naive adult recipients of either RTS,S/AS01 or RTS,S/AS02 were challenged with CHMI, and 31/78 were protected against parasitemia (Figure 1). Transcriptome data were collected from the available archived samples, representing 39 RTS,S recipients, and covered several time points from pre-vaccination to the day of challenge (DOC) at 14 days post-dose 3 (14dPIII). Although that trial estimated efficacies at 50% for RTS,S/AS01 and 32% for RTS,S/AS02, the estimates were not statistically different (*p* = 0.11), probably reflecting the small scale of the trial. To maximize the statistical power of the present study, samples from recipients of either vaccine were considered as a single population. This approach was based on the assumption that by containing the same immunostimulants (MPL and QS-21), AS01 and AS02 have qualitatively similar mechanisms of action.

Multiway partial least squares discriminant analysis was chosen as a data analysis strategy for two main reasons. First, it is capable of analyzing biological changes over time. Second, it can build predictive models on the correlations between class information (in this case protection status) and trends in the data. Therefore, N-PLS-DA contrasts with an earlier analysis of gene expression data from the same clinical trial which considered only a single time point post sporozoite challenge for classification analysis (32, 33). In the present study, N-PLS-DA was applied to the expression patterns of 20,442 microarray probe sets and of selected probe sets of genes in the IFN-pathway. In both approaches, the results were supportive of the involvement of IFN-γ- and NF-κB-signaling pathway genes in the process of establishing RTS,S-mediated protection against CHMI.

## Materials and Methods

### Clinical Trial Samples

PBMCs and serum samples were obtained from participants in the RTS,S vaccine clinical trial (ClinicalTrials.gov NCT00075049) (10).

### Serum IFN-γ and CSP-Specific Antibody Concentrations and Logistic Regression Modeling

Serum IFN-γ was measured using ELISA and protocol from BD Biosciences (Belgium). The assay’s limit of quantification was 1.0 pg/ml. The CSP-specific serum antibody-concentration data have been reported previously (10). Logistic regression modeling using SAS software (Version 9.2, SAS Institute Inc., Cary, NC, USA) was based on classifying CHMI outcome [protected (PR) against parasitemia, non-protected (NP) and, delayed onset of parasitemia (DL; defined as individuals in which parasites were not detected in the blood for a period longer than the longest parasitemia-free period in the control group of infected and unvaccinated subjects)] with the immunogenicity data sets (pre- and post-vaccination time points up to the DOC) and Adjuvant System selected (AS01 or AS02) as covariates. The quality of a model was determined by the area under the curve (AUC) of the receiver operating characteristic (ROC) curve.

### RNA Preparation and Microarray Assay

RNA was prepared from dimethyl sulfoxide-cryopreserved PBMC samples using TRI Reagent and protocol (Molecular Research Center Inc., Cincinnati, OH, USA). The preparation, amplification, and labeling of copy DNA was performed using Ovation RNA Amplification System and protocol (NuGen Technologies, San Carlos, CA, USA). Gene expression levels were determined using Affymetrix HG-U133 Plus 2.0 arrays of 54,120 probe sets and protocol (Affymetrix, Santa Clara, CA, USA). The data were made publicly available *via* ArrayExpress under accession number E-MTAB-4629.

### Microarray Data Normalization

The raw microarray data were normalized *via* GCRMA (34), and outliers were excluded. Values that were within the 99.9% of the estimated distribution of the background expression level were considered background signals. The data from 20,442 probe sets, out of 54,120 probe sets represented on the array, were entered into the analysis based on two criteria: (i) the availability of gene annotation information for the specific probe set and (ii) the presence of a signal larger than background in more than 20% of the arrays. The transcriptome data set was represented as a multiway data set in that it was defined by multiple factors [subject × probe set (gene) × time]. The 3-way matrix data contained 2.05% missing entries, which were handled by the multiway *N*-way toolbox during modeling (35). For the data-driven model, 13 PR and 15 NP subjects were included, and for the IFN-driven model, 12 PR and 15 NP subjects were included. For classification in the data matrices, PR and NP were assigned the values of +1 and −1, respectively. Microarray normalization was performed using the R statistical software (Version 2.11.0) and Bioconductor (Version 2.6).

### Model Selection and Validation

Three techniques of model validation and refinement (36) were used in this study (see the N-PLS-DA Methodological Details section and Figure S1 in Supplementary Material for more details on the methodology); (i) label permutations, (ii) building an ensemble of models (37, 38), and (iii) double cross-validation (DCV) (39, 40), where model performance was determined by the fraction of correctly classified subjects and the DQ^2^ statistic (41). Because the modeling was based on a regression analysis, class value predictions <−1 or >+1 were assigned the values −1 and +1, respectively. In a cross-validation, the subjects of the data set were divided randomly in *N* subsets. One subset was placed aside, and the other subsets were used to build the model. In a DCV, the subjects were divided in *N* subsets and one subset was placed aside. *N* − 1 subsets were again divided into *M* subsets. The subjects in the initially excluded subset were completely independent of the model building and were used to validate the final model. DCV was used for the selection of the number of components and the probe sets to be included in the N-PLS-DA modeling. The different models were evaluated using the DQ^2^ statistic, which is based on a least squares method for analyzing the difference between prediction and CHMI outcome (PR or NP) (41) and was a more discriminatory method for identifying differences in performance than the fraction of correctly classified outcomes. In cases where the optimum number of model refinement rounds was difficult to determine, the fraction of correctly classified outcomes and a statistic based on the mean difference between prediction score and CHMI outcome was also used. Probe sets were selected for evaluation in the DCV based on their individual prediction performance. For the IFN-driven modeling with many fewer probe sets than the data-driven modeling, a forward selection approach was used (42, 43), and model selection only involved the number of components. Label permutations were used to assess whether a model based on the true class labels (i.e., CHMI outcome) performed better than models using the same model parameters but based on randomly assorted class labels. For both the data-driven and IFN-driven modeling, respective summary models were calculated using only the first two components of the transformed data from all of the selected probe sets from the modeling. The components of the summary model were visualized by appropriate rotation on Cartesian axes (with arbitrary units) (44). The N-PLS-DA analyses were performed using Matlab R2010B (MathWorks) the Matlab Statistics Toolbox, and the N-way toolbox (Version 3.1) (35). The biological interpretation of the gene lists was aided by using Ingenuity pathway analysis and upstream transcription factor analysis. The clustering of transcriptome data for heatmap visualization was performed using Cluster 3.0 (45).

## Results

### Serum IFN-γ Concentrations and Vaccine-Mediated Protection Status

In the CHMI clinical trial, three outcomes of sporozoite challenge (after three doses of RTS,S adjuvanted with AS01 or AS02) were defined over the 4-week observation period [Figure 1: protected (PR) against parasitemia, non-protected (NP), and DL (defined as individuals in which parasites were not detected in the blood for a period longer than the longest parasitemia-free period in the control group of infected and unvaccinated subjects)].

**Figure 2:**
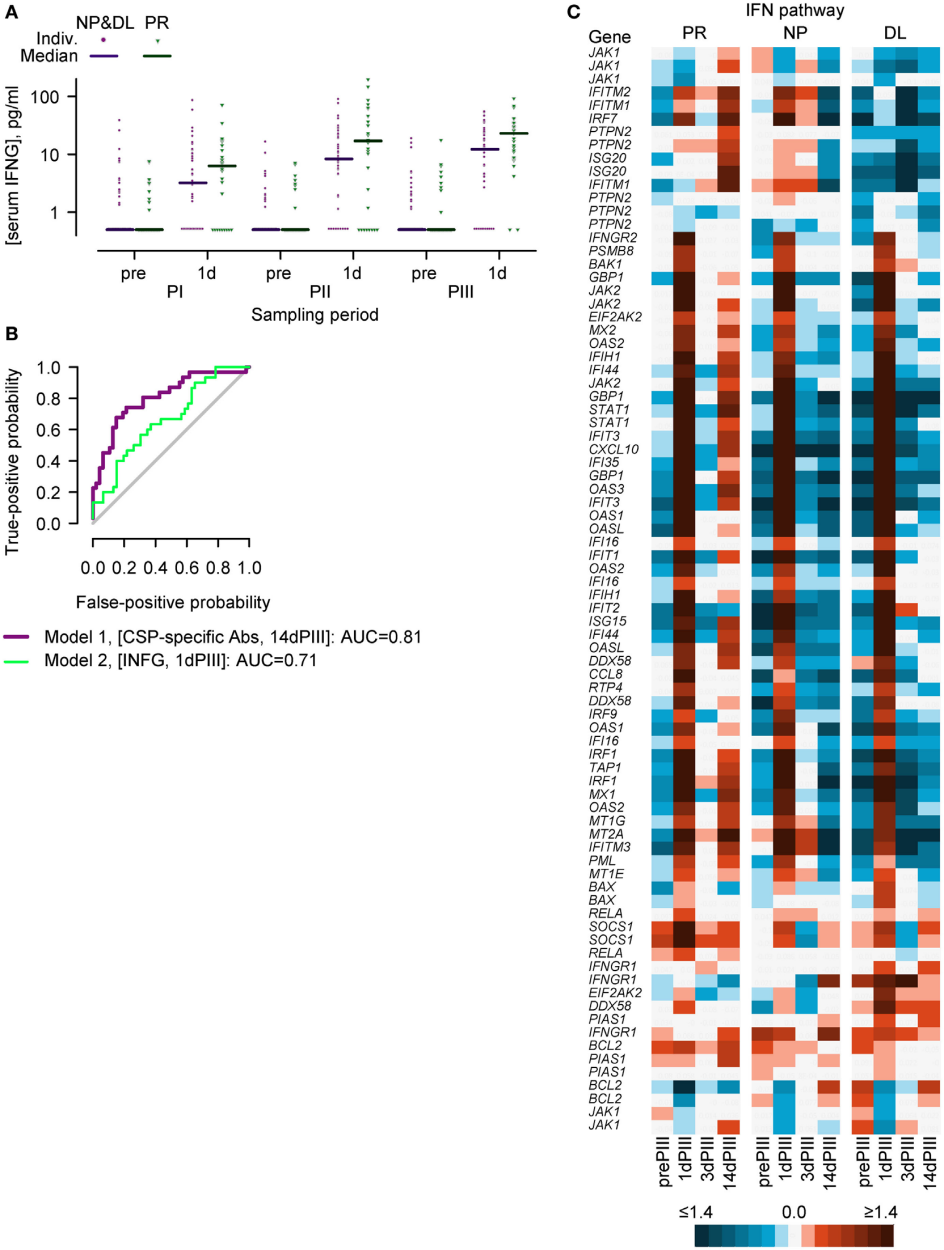
**Serum IFN-γ concentrations and the expression of IFN-pathway genes in PBMCs suggest that the IFN-γ-signaling pathway could play a role in establishing a protective status prior to controlled human malaria infection**. **(A)** Individual and median serum IFN-γ concentrations in protected (PR; *N* = 31) versus non-protected subjects (NP; *N* = 24) plus non-protected subjects with delayed onset of parasitemia (DL, *N* = 23) prior to (pre) and 1 day (1d) after each vaccine dose (PI, PII, or PIII). **(B)** Receiver operating characteristic curves showing the best fit logistic regression model classifying sporozoite challenge outcome (PR versus NP plus DL) with respect to circumsporozoite protein (CSP)-specific antibody concentrations at the day of challenge (14 days post-dose 3) and with respect to IFN-γ concentrations at 1 day post-dose 3 as covariates. **(C)** IFN-pathway gene expression in PR (*N* = 13), NP (*N* = 15), and DL (*N* = 11) groups before (prePIII) and 1, 3, and 14 days after the third vaccine injection (1dPIII, 3dPIII, and 14dPIII, respectively) represented as a heatmap. Mean RNA expression relative to prePI is described in accordance with the colored scale. Certain genes are represented by more than one probe set (see Table S1 in Supplementary Material).

Cell-mediated immunity, including the production of IFN-γ in ELISPOT assays has been associated with RTS,S-mediated protection (10, 20, 21). Moreover, IFN-γ has been detected in the sera of individuals 1 day after the administration of an AS01- or AS02-adjuvanted hepatitis B vaccine candidates and tuberculosis vaccine candidates (46, 47). Given these observations, serum IFN-γ concentrations were measured prior to each dose (prePI, prePII, and prePIII, respectively), and at 1 day after each dose (1dPI, 1dPII, and 1dPIII, respectively) in the entire per-protocol cohort (PPC) (see Figure 1). Higher median concentrations were observed in PR subjects (*N* = 31) than in infected subjects (NP + DL combined, *N* = 47) at 1 day after dose 1 (1dPI; 6.3 versus 3.2 ng/ml), dose 2 (1dPII; 17.1 versus 8.3 ng/ml), and dose 3 (1dPIII; 20.9 versus 12.3 ng/ml; Figure 2A). In logistic regression modeling of sporozoite challenge outcome (PR versus NP and DL) with respect to the immunogenicity data as covariates, the IFN-γ concentrations at 1dPIII could partially explain protection status. The accuracy of the IFN-γ model was estimated at 0.71 from the calculation of the AUC of the ROC graph (Figure 2B; where an accuracy score of 0.5 indicates no accuracy). By comparison, the accuracy score for CSP-specific antibody concentrations at 14 days post-dose 3 (14dPIII; and DOC) was 0.81.

### RNA Expression by IFN-γ-Signaling Pathway Genes in Relation to Protection Status

To further explore the role of IFN-γ in relation to protection against CHMI, transcriptome data were generated from PBMCs isolated from RTS,S vaccinated subjects in the microarray subset (*N* = 39; Figure 1) at prePI and prePIII, and at 1dPIII, 3 days after dose 3 (3dPIII) and 14dPIII. In the microarray subset, the percentages of RTS,S/AS01 recipients over recipients of either adjuvanted vaccine, overall and with respect to each of the post-challenge outcomes, were similar to the respective percentages in the PPC.

With an initial focus on the expression patterns of IFN-pathway genes, an analysis using the Ingenuity pathway analysis tool compared RNA expression patterns in PR and NP subjects. This analysis suggested that in both PR and NP groups, the IFN-γ-signaling pathway was active at 1dPIII (and marked by the increased expression of numerous genes) but not active at 3dPIII (Figure 2C). By contrast, at 14dPIII, the IFN-γ-signaling pathway appeared active in the PR group and not in the NP group.

Although individual PBMC samples were randomly assigned to be processed by one of two batches of microarray kits, the random allocation was not stratified according to protection status, and an imbalance in allocation was observed at 14dPIII. Therefore, by way of validation with respect to a potential batch effect, the expression of the probe sets was examined in a transcriptome data set derived from the same clinical trial, and from a single batch of microarrays (and termed the validation transcriptome) (32). Although the same time points were evaluated, the RNA was not amplified, one less subject was evaluated, and only 74 of the 116 probe sets were present in those microarrays. However, the differences in the patterns of RNA expression in the IFN pathway between PR, NP, and DL groups were similar to that observed in the present transcriptome data set, notably at 14dPIII (see Figure S2 in Supplementary Material that shows the heatmap description of the IFN-pathway gene expression using the validation-transcriptome data set). Therefore overall, the analysis suggested that IFN-γ-signaling could play a role in establishing a protective status prior to sporozoite challenge.

### N-PLS-DA of Transcriptome Data Set in Relation to Protection Status

The transcriptome data set was then further analyzed using N-PLS-DA to identify RNA expression patterns that could distinguish between PR and NP subjects. The method captured the multivariate complexity [subject × probe set (gene) × time] of the data and was used to build mathematical models of correlations between RNA expression data and the binary outcomes of protection or non-protection (see Figure S1 in Supplementary Material that shows a flow diagram description of the N-PLS-DA). Given the onus on requiring a clear binary outcome for the analysis and the complex nature of the data set, the data from the DL subjects were excluded from the modeling. Two approaches were undertaken in the N-PLS-DA that differed by the probe sets that were analyzed. In the first approach, the entire data set was analyzed using N-PLS-DA (data-driven N-PLS-DA), with the aim of obtaining an unbiased selection of genes that could be correlated with protection status. The second approach considered whether IFN-pathway genes alone could be correlated with protection status by only including the data from a selected (i.e., IFN-related) group of probe sets (IFN-driven N-PLS-DA).

Using the data-driven approach, 100 individual models were generated, and each model typically consisted of data from 2 to 40 probe sets. Overall, these models could correctly predict sporozoite challenge outcome on average in 78% of subjects. A total of 116 probe sets were identified on the basis that each probe set was represented at least once in the 100 models. These probe sets corresponded to 110 genes (see Table S1 in Supplementary Material that lists the genes/probe sets selected by the data-driven modeling).

To explore the relationships identified in the 100 models, a summary model was calculated using only the first two components of the transformed data from all of the selected 116 probe sets (consequently, the summary model was not an optimized and validated model because the probe set selection and the number of components were determined outside the model validation context).

**Figure 3:**
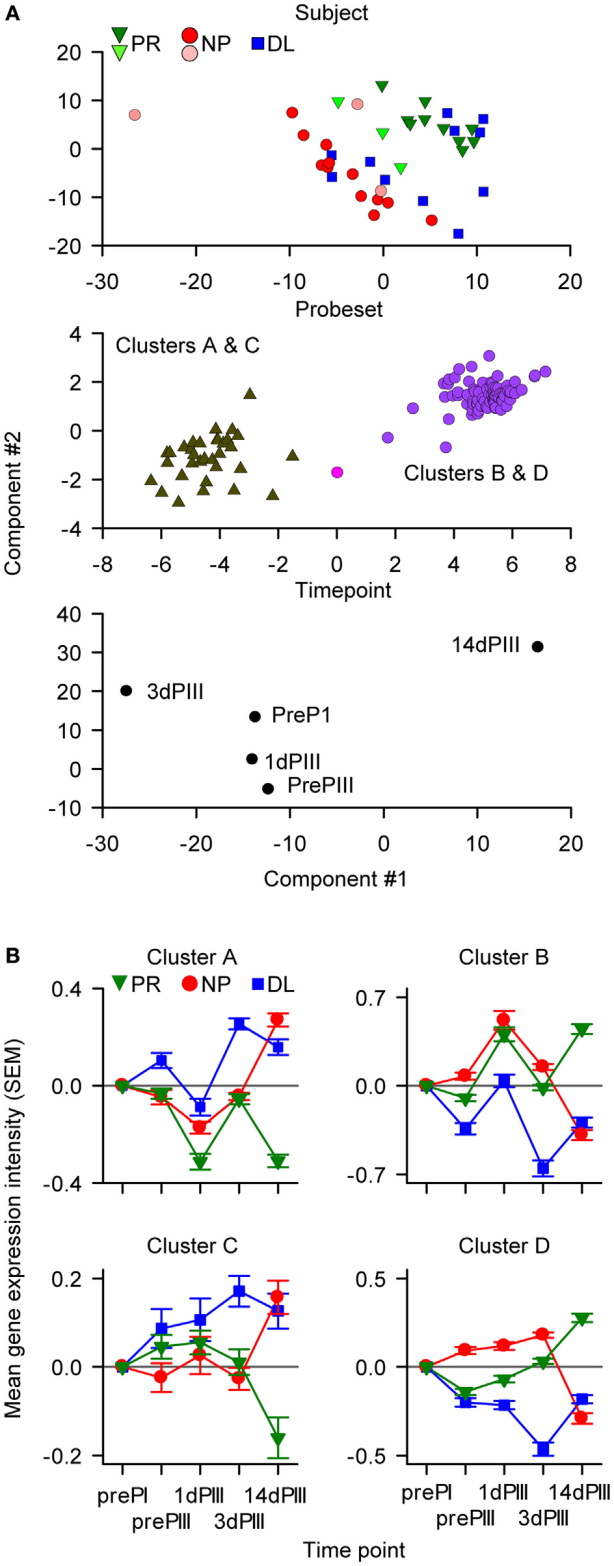
**The data-driven multiway partial least squares discriminant analysis identified 116 probe sets (110 genes) that were classified into four clusters**. **(A)** The components of the summary model were visualized by appropriate rotation on Cartesian axes (with arbitrary units). In the upper graph, data points are distributed with respect to the protection status of subjects. The six data points representing the subjects that were often misclassified in the modeling are represented by the lighter colored symbols. In the middle graph, the data points are distributed with respect to probe set identity into two groups. Each of these two groups comprised two clusters of probe sets (Clusters A and C, and Clusters B and D) based on RNA expression patterns. In the lower graph, the data points are distributed with respect to sampling time [pre-dose 1 (prePI), at pre-dose 3 (prePIII), and 1, 3, and 14 days after dose 3 (1dPIII, 3dPIII, and 14dPIII, respectively)]. **(B)** Mean RNA expression levels relative to prePI, at prePIII and 1dPIII, 3dPIII, and 14dPIII, with respect to protection status of subjects [protected (PR), non-protected (NP), and non-protected with delayed parasitemia (DL)] for each of the four clusters (A–D) of probe sets among the 116 probe sets. The error bars indicate the SEM.

In the summary model and when the data were defined by subject identity, most of the data points could be separated into distinct PR and NP clusters (Figure 3A, upper panel). The N-PLS-DA was done with data from the PR and NP subjects, leaving out the data from the DL subjects. Given that differences were detected between PR and NP subjects, it was of interest to study the data from DL subjects with respect to the two clusters. Hence, when the summary model was imposed on the data from the DL subjects, the data points were distributed among both of the PR and NP clusters. The data from six subjects (three PR and three NP; and three each from AS01 and AS02 groups, respectively) who were often misclassified in the prediction rounds during model validation were mostly positioned close the boundary between PR and NP clusters. When the data were defined by probe set identity, two distinct clusters could be identified by applying *post hoc k*-means clustering (48) (Figure 3A, middle panel) and further analysis revealed that these two clusters corresponded to two different types of expression kinetics in PR subjects or in NP subjects (see below). When the data were defined by sampling time point (Figure 3A, lower panel), the dispersion between consecutive time points was larger for 1dPIII to 3dPIII and 3dPIII to 14dPIII data points than for prePI to prePIII and prePIII to 1dPIII data points suggesting that the 3dPIII and 14dPIII time points contributed most to predicting the distinction between PR and NP in the modeling.

Using additional *k*-means clustering, four distinct clusters of probe sets with different expression-kinetic profiles were identified (Figure 3B and see Figure 3A, middle panel; Table S1 in Supplementary Material). This clustering was performed on the RNA expression values of the 116 probe sets from the PR subjects at different time points relative (by subtraction with log-transformed values) to the prePI time point. The same clustering was then imposed on NP and DL data sets (Figure 3B). The greatest divergence in the expression kinetics between the PR and NP groups occurred between 3dPIII and 14dPIII. The directionality of this divergence for Clusters A and C probe sets was opposite to that for Clusters B and D probe sets. For Clusters A and C probe sets, expression levels decreased in the PR group and increased in the NP group between 3dPIII and 14dPIII, whereas the reverse was observed for Clusters B and D probe sets. Interestingly, although the expression levels for the DL group at 14dPIII were similar to those of the NP group, the directionality of expression kinetics for the DL group between 3dPIII and 14dPIII was similar to those of the PR group, suggesting that the selected 116 probe sets may be able to distinguish the DL outcome from both PR and NP outcomes.

### IFN-γ-Pathway Genes Feature among the Genes Identified by the Data-Driven N-PLS-DA

**Figure 4:**
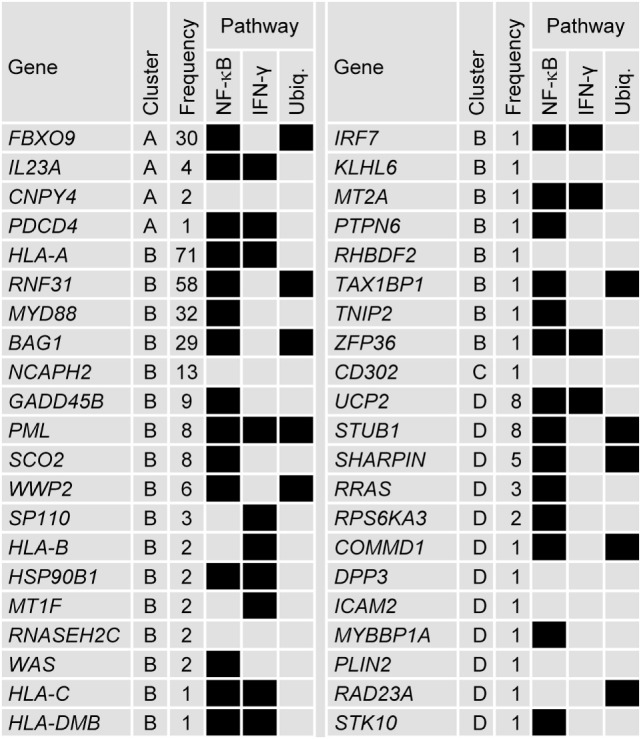
**Forty-two genes selected by the data-driven modeling were immune-related**. Tabular description of the association between the 42 immune-related genes selected by the data-driven modeling and NF-κB, IFN-γ, and ubiquitination (Ubiq.) pathways (associations represented by black boxes). For each gene, the cluster allocation and the frequency it represented in the modeling process is indicated. For genes that were represented by more than one probe set, the data for the most frequently represented probe set are shown (see Table S1 in Supplementary Material). The references supporting the immune-related associations are detailed in Table S2 in Supplementary Material.

Of the 110 genes represented in the 100 models, 42 have been previously ascribed immune-related functions, of which 13 were represented at least five times in the models (Figure 4; see Tables S1 and S2 in Supplementary Material that lists the references to support the characterization of the immune-related genes selected by the data-driven modeling). The immune-related genes were most frequently identified in Cluster-B (i.e., 25/42 genes) including two (*HLA-A* and *RNF31*) of the three most frequently represented genes. Fourteen immune-related genes have been associated with regulating IFN-γ signaling or with being downstream targets of IFN-γ signaling. These latter genes include the major histocompatibility complex (MHC) class I and class II genes. Twenty-nine immune-related genes have been associated with the NF-κB pathway, including upstream regulators such as the frequently represented gene *MYD88*, a key mediator of TLR and IL1-R signaling. Ten immune-related genes (including frequently represented genes *RNF31, BAG1, FBXO9, PML, STUB1, WWP2*, and *SHARPIN*) have been associated with regulating ubiquitination, nine of which have been also associated with the NF-κB pathway. Genes such as *STUB1, PLIN2, PML, HSP90B1*, and *RAD23A*, in addition to the MHC class I genes, have been associated with the antigen presentation pathway. Two frequently represented genes (*NCAPH2* and *GADD45B*) have been associated with T-cell and B-cell survival, respectively. For the other 69 genes, immune-related functions have not been described. Nineteen of these genes were represented at least five times including *GTF2E2* and *GTF2F1*, which were the first and fifth most frequently represented genes overall, respectively, and code for subunits of the general transcription factor complex that is essential for transcriptional initiation.

**Figure 5:**
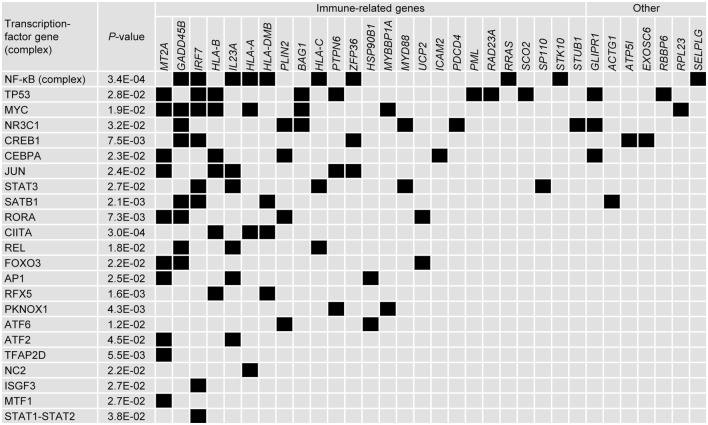
**Upstream *in silico* analysis suggests that numerous immune-related genes can be placed within networks defined by common transcription factors**. Tabular representation of the upstream transcription factors that were associated with the genes selected by the data-driven modeling (associations represented by black boxes). Transcription factors were selected using Ingenuity, in which associations were also ascribed *p*-values. Transcription factors and were ranked by number of associations and genes were ranked by classification (immune-related/other) and number of associations.

Further connections between genes were analyzed by identifying common upstream transcription factors. Thirty two of the 110 genes were allocated to one or several of 23 groups defined by a common upstream transcription factor (or complex) (Figure 5). Twenty-two of the genes identified were immune-related and each group contained at least one immune-related gene. As expected, the NF-κB complex of transcription factors was identified as regulating several genes. Other immune-related transcription factors were also identified such as CIITA, RFX5, and SATB1 [that regulate *HLA* genes (49, 50)], NR3C1 [glutocorticoid receptor (51, 52)] and REL [part of the NK-κB complex (53, 54)]. Although transcriptional regulators such as TP53, MYC, CREB1, STAT3, and CEBPA have been implicated in numerous pathways, they have also been associated with regulating innate immune pathways (55–63).

The expression patterns of the probe sets from the different clusters were also examined in the validation-transcriptome data set. A clear distinction between PR and NP subjects was observed for Cluster-B probe sets (see Figure S3 in Supplementary Material that shows the expression of Clusters A–D probe sets using the validation-transcriptome data set), with a divergence in expression kinetics from 3dPIII to 14dPIII that was similar to that in the present transcriptome data set, suggesting that the differences in expression of Cluster-B probe sets with respect to protection status were not masked by a potential batch effect at 14dPIII. However, whether batch effect abrogated differences in expression patterns for the probe sets from the other clusters could not be concluded because of the potentially inferior quality of validation-transcriptome data (e.g., absence of an RNA amplification, fewer probe sets).

### Predictive Models of Protection Can Be Built from Probe Set Data Representing IFN-γ-Signaling Pathway

**Figure 6:**
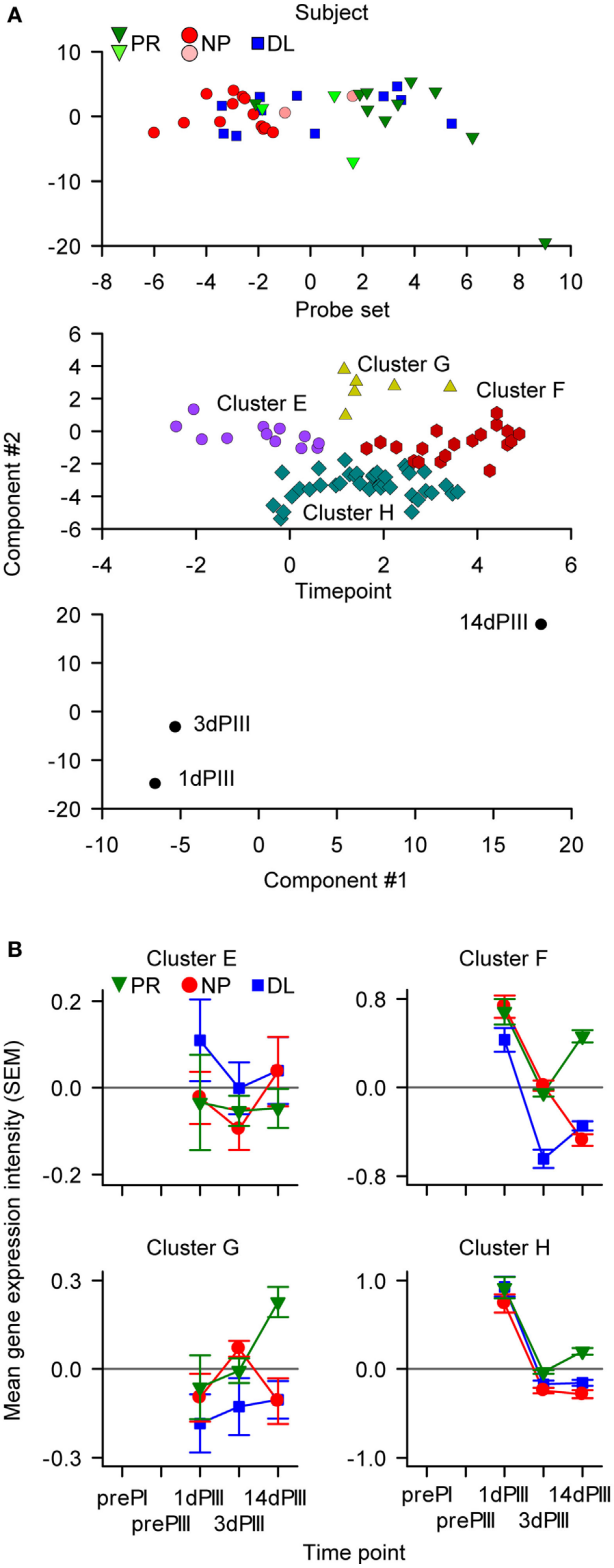
**The IFN-driven multiway partial least squares discriminant analysis identified 74 probe sets (44 genes) classified into four clusters**. **(A)** The components of the summary model were visualized by appropriate rotation on Cartesian axes (with arbitrary units). In the upper graph, data points are distributed with respect to the protection status of subjects. The six data points representing the subjects that were often misclassified in the modeling are represented by the lighter-colored symbols. In the middle graph, the data points are distributed into four clusters of probe sets (Clusters E–H). In the lower graph, the data points are distributed with respect to sampling time [only 1, 3, and 14 days after dose 3 (1dPIII, 3dPIII, and 14dPIII, respectively) were considered in the modeling]. **(B)** Mean RNA expression levels relative to prePI, at 1dPIII, 3dPIII, and 14dPIII, with respect to protection status of subjects [protected (PR), non-protected (NP), and non-protected with delayed parasitemia (DL)] for each of the four clusters (E–H) of probe sets among the 74 probe sets. The error bars indicate the SEM.

Based on the links between the IFN-γ secretion data (Figure 2A) and IFN-γ-signaling pathway that emerged from the pathway analysis (Figure 2C) and the data-driven N-PLS-DA (Figure 4), further modeling was performed with a data set restricted for genes associated with the IFN pathway. Thus, 45 genes, represented by 82 probe sets (see Table S3 in Supplementary Material that lists the genes/probe sets used in the IFN-driven modeling), were assigned to the N-PLS-DA model-building process (IFN-driven N-PLS-DA; see Figure S1 in Supplementary Material). From this process, 74 probe sets (44 genes) were represented at least once in the 100 models (Table S3 in Supplementary Material). The models could correctly predict protection on average in 77% of subjects. A summary model was generated using data from the three time points (1dPIII, 3dPIII, 14dPIII) because the post-Dose 3 time points appeared to best capture differences between the PR and NP groups in the data-driven N-PLS-DA analysis. In the summary model, similar patterns emerged to those found with the data-driven N-PLS-DA. When the data were defined by subject identity, a clear distinction between PR and NP subject data points was identified (Figure 6A, upper panel), in which DL-subject data points were allocated to both of PR and NP clusters. The data points from five subjects (three PR and two NP; and two from the AS01 group and three from the AS02 group) who were often misclassified in the prediction rounds during model validation were mostly positioned close to the boundary between PR and NP clusters. The data points from three of these subjects were also misclassified in initial N-PLS-DA. When the data were defined by probe set identity, four clusters (Clusters E–H) were identified with different kinetic expression profiles (Figure 6A, middle panel, and Figure 6B). Most probe sets were assigned to Clusters F and H. When the data were defined by time point, the greater separation between consecutive time points was from 3dPIII to 14dPIII rather than 1dPIII to 3dPIII (Figure 6A, lower panel).

As with the data-driven selected probe sets, the greatest divergence in the RNA expression kinetics between the PR and NP groups for the IFN-pathway probe sets, occurred from 3dPIII to 14dPIII (Figure 6B). At 1dPIII, expression levels were high for Clusters F and H, and in contrast to near-zero RNA expression levels (i.e., baseline) for Cluster E and G (Figure 6B). Interestingly, in terms of divergence in the kinetics between the PR and NP groups from 3dPIII to 14dPIII, the expression kinetics for Clusters F and H were similar to those for Cluster-B in the data-driven N-PLS-DA (see Figure 4). As with the data-driven selected probe sets, the expression levels for the DL group at 14dPIII were similar to those for the NP group for Cluster E, F, G, and H probe sets, whereas only for Cluster-F probe sets were the directionalities of expression kinetics for the DL group from 3dPIII to 14dPIII similar to those of the PR group. Therefore, this suggested that the Cluster-F probe sets may be able to distinguish the DL outcome from both PR and NP outcomes.

The most frequently represented gene by the 74 probe sets in the modeling was *TAP1*, appearing 79 times (out of 100 models). *TAP1* and other frequently represented genes such as *JAK1* (67 times) *STAT1* (44 times), *IRF1* (36 times) form a core part of the IFN-γ-signaling pathway, and exemplify genes for which expression levels at 14dPIII (relative to prePI) were distinctly different between the PR and NP groups, in contrast to only minor differences in expression levels at 1dPIII. Moreover, *TAP1, STAT1*, and *IRF1* were represented by Cluster-F probe sets and therefore may capture differences between the DL outcome from both PR and NP outcomes. The frequently represented genes *MT2A* (66 times), *IRF7* (36 times), and *PML* (8 times) were also identified in the data-driven N-PLS-DA.

## Discussion

Systems biology analyses are potentially advantageous in clinical trial settings where multiparametric data are often generated (28, 64, 65). Signals from both innate and adaptive immune responses can be identified in transcriptome data derived from peripheral blood from 3 days after vaccination (25, 26). Here, we have identified two gene signatures by modeling longitudinal PBMC-transcriptome data from clinical trial subjects who were vaccinated with RTS,S/AS01 or RTS,S/AS02 and then challenged with *P. falciparum* sporozoites. In having an efficacy endpoint in the vaccine trial, our study makes an advance on earlier systems biology analyses of other vaccine trials in which only immunogenicity endpoints were considered (25–27, 66–68).

Our study was hypothesis generating in its design, given that the gene signatures were not validated in an independent and similarly designed clinical trial. Also, the interpretation of the systems biology analyses was limited by three principal factors. First, the samples were derived from peripheral blood and not at the injection site or draining lymph nodes where most of the direct responses to the vaccine components would have occurred. Second, the samples contained a heterogeneous population of cells, and hence the variation in gene-expression levels may have arisen from relative differences in cell population sizes or from relative differences in a given cell population. Third, to increase the statistical power, the samples from recipients of either AS01- or AS02-adjuvanted vaccines were considered as a single population based on the assumption that by containing the same immunostimulants (MPL and QS-21), AS01 and AS02 have qualitatively similar mechanisms of action.

The two gene signatures were identified by applying N-PLS-DA both to the entire transcriptome data set and to data from selected genes within the IFN-γ-signaling pathway. In the first data-driven approach, a gene signature, consisting of 110 genes including 42 known immune-related genes was identified. Of the immune-related genes, many were associated with the IFN-γ and NF-κB pathways; and a third of the genes associated with the NF-κB pathway were also associated with regulating ubiquitination. Hence, the validity of the gene signature was supported by the functional connectivity of many of the immune-related genes. Other selected genes may have immune-related functions but these remain to be characterized. For example, genes such as those of the general transcription factor complex (*GTF2E2* and *GTF2F1*) may have been selected because they may have a prominent function in immune-related cell populations. The second approach confirmed that a gene signature based on IFN-pathway genes alone could have a similar predictive capacity to that of the data-driven gene signature because 44 of the 45 *a priori* selected genes were included in the modeling. Overall, the differences in serum IFN-γ concentrations and the identities of the two gene signatures suggested that the IFN-γ pathway plays a role in RTS,S vaccine-mediated protection. The gene signatures may have captured differences between subjects in terms of responses to the Adjuvant System (AS01 or AS02) because these Adjuvant Systems appear to better enhance IFN-γ-related CMI responses to vaccination in comparison with other adjuvants (20, 69).

The predictive power of the N-PLS-DA modeling was around 78% because in the two approaches, the models often misclassified four NP and four PR subjects (i.e., 16–19%). This suggests that the transcriptome data alone was inadequate in these cases to make accurate predictions, perhaps because of other unknown confounding factors. In the primary analysis of the clinical trial, a similar proportion of individuals (8/51, 16%; 5P and 3NP), but not all the same individuals, were misclassified based on assignments decided by threshold DOC antibody titers (10).

Multiway partial least squares discriminant analysis contrasts with other transcriptome data analysis in that it can incorporate both the kinetics of the immune response and the inherently multivariate nature of transcriptome data. Our conclusions extend previous transcriptome data analyses of the same vaccinated cohort, in which individual time points were considered separately (32, 33). Rinchai et al. (33) identified differences in gene expression between PR and NP subjects at 1dPIII, 3dPIII, and 14dPIII using a predefined cluster of 130 probe sets (Module 1.2) that had previously been shown to reflect type-1 interferon signaling (68). Vahey et al. (32) identified the immunoproteasome pathway using a supervised approach [Gene Set Enrichment Analysis (GSEA)] on gene-expression data at 14dPIII (DOC). Although Module 1.2 or Cluster-B (in our study) did not include probe sets representing those immunoproteasome genes, the immunoproteasome is regulated by IFN-γ (70–72), and therefore, the identification of those genes may have reflected differences in IFN-γ signaling between PR and NP subjects. Furthermore, one Cluster-B gene, *PML*, has been identified as a regulator of the immunoproteasome (73). Also, the IFN-driven N-PLS-DA confirmed that genes related to the immunoproteasome [i.e., *TAP1* (74) and *PSMB8* (*LMP7*) (70)] can be incorporated into the predictive models.

The trends in the gene expression kinetics suggested that differences between PR and NP subjects was most notable with the divergence of the expression kinetics after 3dPII leading to clear differences in relative expression levels at 14dPIII for the four clusters of the data-driven gene signature and Cluster-F of the IFN-driven gene signature. Furthermore, the expression kinetics appeared to distinguish the DL outcome from both the PR and NP outcomes, in that the trajectory of the expression kinetics in DL subjects after 3dPIII were similar to those in PR subjects. Surprisingly, the differences between PR and NP groups in terms of expression levels of the clusters of IFN-driven signature genes was not apparent at 1dPIII and 3dPIII, even though the differences in serum IFN-γ concentrations between PR and NP groups at 1dPIII appeared to contribute toward protection. Rather, the expression data suggested that the IFN-γ pathway was transiently activated in the peripheral circulation in all groups, but signs of its reactivation occurred primarily in PR subjects, at 14dPIII.

Therefore, our hypothesis is that the gene signatures reflect the presence of activated effector/effector memory (E/EM) CD4^+^ T cells that are induced after immunization and are responsible for IFN-γ signaling in a different manner in PR subjects to that in NP subjects. The potential role of CD4^+^ T cells in the release of IFN-γ into the serum is suggested by the observed increase in the serum IFN-γ concentrations after repeated vaccination in this study and in a previous study of AS01- and AS02-adjuvanted hepatitis B vaccines (46). Although it is possible that antigen-specific CD4^+^ or CD8^+^ T cells were directly responsible for IFN-γ production, this cytokine was not prominently produced by antigen-specific CD4^+^ T cells, and antigen-specific CD8^+^ T cells were not detected in PBMCs after vaccination in the same clinical trial (10, 23). Alternatively, other cells, such as NK cells, may have produced IFN-γ. NK cells have been shown to produce IFN-γ in response to signals from antigen-specific CD4^+^ T cells (75–77) and from other innate immune cells activated by AS01 in the lymph nodes draining the vaccine injection site (Margherita Coccia, unpublished data). Given that antigen-specific CD4^+^ T cells can stimulate IFN-γ production in NK cells in an IL-2-dependent manner (75–77), it is notable that in a previous analysis of the same clinical trial, the frequencies of IL-2 producing T_E/EM_ and central memory T cells in PR subjects were significantly higher than in NP subjects (23). Also CD4^+^ T cell-mediated activation of NK cells may depend on CD40–CD40L interactions (78) as well as NF-κB signaling (79). Another intriguing possibility is that NK cells may be differently primed in PR versus NP individuals by the time of the third RTS,S dose, thus contributing to the differences in IFN-γ production after the third dose. Kazmin et al. performed a systems biology analysis (80) of a more recent CHMI efficacy trial of RTS,S (19), in which individual time points pre- and post-vaccination were considered, and which used the same validation-transcriptome data set as our study. In their study, several models predictive of protection were identified at prePIII (the day of the third RTS,S dose). The frequently represented genes in those models and other GSEA analyses identified an inverse correlation between NK-cell-related gene expression in the blood and protection, suggesting that in those individuals who were subsequently protected, there was a greater efflux of NK cells from the blood expressing homing receptors to the draining lymph node or injection site between the second and third RTS,S doses.

Hence, an enticing corollary to our hypothesis is that the interactions between NK cells, CSP-specific antibodies, and CD4^+^ T cells may be directly relevant in the subsequent clearance of sporozoite-infected hepatocytes through mechanisms such as antibody-mediated cell cytotoxicity. A role of IFN-γ produced by NKT cells to suppress sporozoite-infected hepatocytes was recently described in a mouse model (81). It is possible, therefore, that RTS,S-induced memory responses were capable of mobilizing NKT cells or other sources of IFN-γ production in the protective response to sporozoite challenge. Furthermore, and despite the hypothesis-generating nature of our study, the genes identified by the systems biology analyses shed useful light on understanding how protection against malaria parasitemia is achieved by RTS,S.

## Ethics Statement

The clinical trial was conducted in accordance with all applicable regulatory requirements, including the Declaration of Helsinki (1996). The clinical trial was approved by institutional review boards from the Walter Reed Army Institute of Research (WRAIR) Human Use Review Committee (HURC) and the United States Army Medical Research and Materiel Command (USAMRMC) Human Subjects Research Review Board (HSRRB). Written-informed consent was obtained from each participant prior to the performance of any study-specific procedures in accordance with relevant ICH Guidelines, US Army Regulations, and principles of the Declaration of Helsinki.

## Author Contributions

WRB, KK, and CO participated in the design and conduct of the clinical trial from which the samples were acquired. RB, JV, EJ, and RM conceived, and/or designed and implemented the transcriptome data analyses. RB, RM, AD, and MC conceived, designed, and implemented the immunogenicity logistical-regression analysis. All authors contributed to the interpretation of the results, as well as to the development of this manuscript. All authors had full access to the data and approved the manuscript before it was submitted by the corresponding author. The opinions or assertions contained herein are the private views of the authors and are not to be construed as official of as reflecting the views of the United States Department of the Army or the Department of Defense.

## Conflict of Interest Statement

All authors declared the following interests: RB, MC, WRB, AD, EJ, and RM are employees of the GSK group of companies. JV was an employee of the GSK group of companies at the time of the study but is now affiliated with WHO, which was not involved in the study. The opinions or assertions contained herein are not to be construed as reflecting the views of the WHO. RB, WRB, AD, EJ, and RM report ownership of GSK shares and/or restricted shares. KK is an employee of Sanofi Pasteur. CO declares no competing financial interests.

## Abbreviations

1dPI, 1dPII, and 1dPIII, 1 day after dose 1, dose 2, and dose 3, respectively; 3dPI and 14dPIII, 3 and 14 days after dose 3, respectively; AUC, area under the curve; CHMI, controlled human malaria infection; CMI, cell-mediated immunity; CSP, circumsporozoite protein; DCV, double cross-validation; DL, delayed onset of parasitemia; DOC, day of challenge; GSEA, Gene Set Enrichment Analysis; NP, non-protected against parasitemia; N-PLS-DA, multiway partial least squares discriminant analysis; PR, protected against parasitemia; prePI, prePII, and prePIII, prior to dose 1, dose 2, and dose 3, respectively; ROC, receiver operating characteristic.
