# Supplementary material for: Predicting RTS,S Vaccine-Mediated Protection from Transcriptomes in a Malaria-Challenge Clinical Trial
Source: Front Immunol. 2017 May 23;8:557. doi: 10.3389/fimmu.2017.00557 (PMC5440508; doi:10.3389/fimmu.2017.00557)
Supplement: Supplementary file 1 [file table_1.pdf]

Supplementary Material

Predicting RTS,S-vaccine-mediated protection from transcriptomes in a malaria-challenge clinical trial

Robert A. van den Berg,* Margherita Coccia, W. Ripley Ballou, Kent E. Kester, Christian F. Ockenhouse, Johan Vekemans, Erik Jongert, Arnaud M. Didierlaurent, Robbert van der Most.

*Address correspondence to: Robert A. van den Berg, GSK Vaccines, Rue de l'Institut, 89, 1330 Rixensart, Belgium. Telephone, +3226568395; E-mail, robert.x.van-den-berg@gsk.com.

1 N-PLS-DA METHODOLOGICAL DETAILS

The transcriptome data set was represented as a multiway data set (subject×probe set [gene]×time). In the N-PLS-DA, the data were transformed in to a series of components (similar to principal component analysis [PCA] (Jackson, 1991;Jolliffe, 2002)) where the first component encapsulates the most variation in the total data set that correlates with controlled human malaria infection (CHMI) outcome, and the subsequent components encapsulate progressively less variation. Together, these factors describe variations in the data set which were encapsulated in predictive mathematical models. Hence the kinetics of the changes induced by the vaccination were captured explicitly in each of the mathematical models.

Each mathematical model was generated through the iterative selection of probe sets and the selection of the minimal number of components required from the transformed data set to achieve optimal model performance. Model performance was evaluated by a double cross validation (DCV) approach. DCV resulted in 10 collections (ensembles) of 10 models of correlation, yielding a total of 100 individual models, with performance statistics. The difference in model performance was identified using the DQ^2 statistic. This statistic is based on a least-squares method for analyzing the difference between prediction and CHMI outcome (Westerhuis et al., 2008) and was more discriminatory than using the fraction of correctly classified outcomes. The consideration of two or three components was typically sufficient for optimal prediction performance. Each model typically consisted of data from 2-40 probe sets and optimal performance was typically observed after several rounds of probe set selection. Predictive performance was validated using label permutation. The worst, average and best model performance measures in a given ensemble of models were always higher than the most frequent performance measure generated by label

REFERENCES

Jackson, J.E. (1991). *A user's guide to principal components.* New York, NY: John Wiley & Sons Inc.

Jolliffe, I.T. (2002). *Principal component analysis.* New York, NY: Springer-Verlag.

Westerhuis, J.A., Van Velzen, E.J., Hoefsloot, H.C., and Smilde, A.K. (2008). Discriminant Q^2 (DQ^2) for improved discrimination in PLSDA models. *Metabolomics* 4**,** 293-296.

2 SUPPLEMENTARY TABLE 1

Genes/probe sets selected by the data-driven modeling

Gene	Probe set ID	Frequency of use in models	Cluster
ACTG1	224585_x_at	1	D
ATL2	222700_at	2	A
ATP5I	209492_x_at	4	D
ATP6V0C[a,b]	36994_at	20	D
ATP6V0C[a]	200954_at	1	D
AURKAIP1	225555_x_at	8	D
B3GALT6	1553959_a_at	1	D
BAG1	202387_at	29	B
BAG5	202984_s_at	3	D
C11orf73	219979_s_at	1	C
C14orf64	1559097_at	3	A
CCDC59	222792_s_at	2	D
CCT6P1	227301_at	1	A
CD302	203799_at	1	C
CNPY4	227313_at	2	A
COMMD1	226024_at	1	D
CSNK1G2	202573_at	10	D
DENND4C	205684_s_at	1	A
DPP3	232510_s_at	1	D
DVL1	203230_at	1	C
EDEM2	78047_s_at	5	D
EIF4E2	213571_s_at	11	B
EMC10	224727_at	21	B
EXOSC6	227696_at	3	A
FAM21A/B /C	212370_x_at	4	B
FAM21A/B /C /D	214946_x_at	1	B
FAM53C	218023_s_at	1	B
FBXO9	212991_at	30	A
GADD45B	207574_s_at	9	B
GLIPR1	226142_at	1	D
GTF2E2	202680_at	97	B
GTF2F1	202355_s_at	45	D
HECTD1	224481_s_at	2	A

Gene	Probe set ID	Frequency of use in models	Cluster
HLA-A[a,b]	215313_x_at	71	B
HLA-A[a]	213932_x_at	1	B
HLA-B	209140_x_at	2	B
HLA-C	208812_x_at	1	B
HLA-DMB	203932_at	1	B
HSP90B1	200598_s_at	2	B
ICAM2	213620_s_at	1	D
IDH3G	202471_s_at	6	B
IL23A	217328_at	4	A
IPW	241834_at	1	A
IRF7	208436_s_at	1	B
KLHL6	1560396_at	1	B
LMLN	244881_at	3	A
LRPAP1	201186_at	11	D
LRRC14	32062_at	1	D
LSMEM1	239203_at	2	C
LYRM2	227712_at	6	D
MRFAP1	226091_s_at	1	C
MT1F	217165_x_at	2	B
MT2A	212185_x_at	1	B
MYBBP1A	219098_at	1	D
MYD88	209124_at	32	B
NAP1L5	228062_at	1	A
NAPRT1	226707_at	1	B
NCAPH2	40640_at	13	B
NCBP2-AS2	225657_at	9	D
NCF1C	214084_x_at	1	B
NOB1	223018_at	57	D
NSD1	219084_at	2	B
NUCKS1	229353_s_at	1	D
NUDT14	231914_at	2	D
OR2A9P	222290_at	10	A
ORC2	204853_at	11	A
PDCD4	212593_s_at	1	A
PDZK1	205380_at	1	A
PLA2G12A	242323_at	2	A
PLIN2	209122_at	1	D

Gene	Probe set ID	Frequency of use in models	Cluster
PMF1	202337_at	2	D
PML	211012_s_at	8	B
POLE4	1553587_a_at	1	B
PTPN6	206687_s_at	1	B
PUS7L	229751_s_at	3	A
RAD23A	201046_s_at	1	D
RAPH1	225189_s_at	1	A
RBBP6	227635_at	5	B
RHBDF2	219202_at	1	B
RIC8A	221647_s_at	2	D
RMND5A	212482_at	4	A
RNASEH2C	226453_at	2	B
RNF31	231635_x_at	58	B
RPF2	225866_at	1	D
RPL23	200888_s_at	7	C
RPS6KA3	203843_at	2	D
RRAS	212647_at	3	D
SCO2	205241_at	8	B
SELPLG	209879_at	1	D
SFXN3	217226_s_at	1	D
SHARPIN	220973_s_at	5	D
SP110[a,b]	223980_s_at	3	B
SP110[a]	209762_x_at	2	B
SRSF1	211784_s_at	1	C
STK10	40420_at	1	D
STUB1	217934_x_at	8	D
SWT1	223548_at	1	A
TAF1	227205_at	1	A
TAF10	200055_at	1	D
TAX1BP1	200977_s_at	1	B
TBCB[a,b]	211759_x_at	17	D
TBCB[a]	216194_s_at	1	D
TNIP2	48531_at	1	B
TRAPPC5	225870_s_at	1	D
TRIM26	202702_at	4	B
TRIM52	1568594_s_at	1	A
TSTA3	36936_at	1	D

Gene	Probe set ID	Frequency of use in models	Cluster
TTC33	231479_at	1	A
UBB	200633_at	2	D
UCP2[a,b]	208997_s_at	8	D
UCP2[a]	208998_at	8	D
USP40	225089_at	1	A
WAS	38964_r_at	2	B
WWP2	204022_at	6	B
ZFP36	201531_at	1	B
ZNF117	235408_x_at	9	C

[a]For genes with more than one probe set, the data from [b]the probe set that was most frequently represented in the models were considered as the representative data for that gene in the manuscript.

3 SUPPLEMENTARY TABLE 2

References to support the characterization of the immune-related genes selected by the data-driven modeling.

Gene	Cluster	Freq.	NF-κB	IFN-γ	Ubiq.	References
FBXO9	A	30	+		+	(Cenciarelli et al., 1999;Winston et al., 1999;Yang et al., 2009)
IL23A	A	4	+	+		(Carmody et al., 2007;Teng et al., 2010;Qian et al., 2011;Sheikh et al., 2011)
CNPY4	A	2				(Konno et al., 2006;Hart and Tapping, 2012)
PDCD4	A	1	+	+		(Hilliard et al., 2006;Yamanaka et al., 2009;Sheedy et al., 2010;Cohen and Prince, 2013)
HLA-A	B	71	+	+		(Hakem et al., 1991;Girdlestone et al., 1993;Min et al., 1996;Boehm et al., 1997;Girdlestone, 2000;Johnson, 2003;Shen et al., 2009;Othman et al., 2012;Norman et al., 2013;Sleiman et al., 2014)
RNF31	B	58	+		+	(Gerlach et al., 2011;Ikeda et al., 2011;Tokunaga et al., 2011;Stieglitz et al., 2013;Schaeffer et al., 2014)
MYD88	B	32	+			(Adachi et al., 1998;Burns et al., 1998;Cohen, 2014)
BAG1	B	29	+		+	(Tsukahara and Maru, 2010;Kettern et al., 2011)
NCAPH2	B	13				(Gosling et al., 2007;Gosling et al., 2008)
GADD45B	B	9	+			(Takekawa and Saito, 1998;Papa et al., 2004;Liu et al., 2005;Thyss et al., 2005)
PML	B	8	+	+	+	(Wu et al., 2003;Carracedo et al., 2011;Guo et al., 2014)
SCO2	B	8	+			(Mauro et al., 2011)
WWP2	B	6	+		+	(Chen et al., 2009;Yang et al., 2013)
SP110	B	3		+		(Pan et al., 2005;Tosh et al., 2006;Abhimanyu et al., 2011;Cai et al., 2013;Fox et al., 2014)
HLA-B	B	2		+		(Chamberlain et al., 1991;Hakem et al., 1991;Min et al., 1996;Boehm et al., 1997)
HSP90B1	B	2	+	+		(Randow and Seed, 2001;Yang et al., 2007;Liu et al., 2010;Staron et al., 2010)
MT1F	B	2		+		(Vandeghinste et al., 2000;Huang et al., 2009)
RNASEH2C	B	2				(Rice et al., 2013;Crow et al., 2015)

Gene	Cluster	Freq.	NF-κB	IFN-γ	Ubiq.	References
WAS	B	2	+			(Orange et al., 2002;Borg et al., 2004;Gismondi et al., 2004;Huang et al., 2005;Krzewski et al., 2006;Serrano-Pertierra et al., 2012;Ham et al., 2013;Catucci et al., 2014;Sarkar et al., 2014;2015)
HLA-C	B	1	+[a]	+		(Boehm et al., 1997;Boss, 1997;Johnson, 2003;Shen et al., 2009;Othman et al., 2012)
HLA-DMB	B	1	+[a]	+		(Steimle et al., 1994;Boehm et al., 1997;Boss, 1997;Westerheide et al., 1997)
IRF7	B	1	+	+		(Kawai et al., 2004;Ogawa et al., 2005;Chau et al., 2008;Beattie et al., 2011)
KLHL6	B	1				(Gupta-Rossi et al., 2003;Kroll et al., 2005)
MT2A	B	1	+	+		(Giacconi et al., 2007;Jin et al., 2010;Toh et al., 2010;Pan et al., 2013)
PTPN6	B	1	+			(Tsui et al., 2006;Orr et al., 2010;Lee et al., 2011;Orr and Lanier, 2011;Wang et al., 2012a)
RHBDF2	B	1				(Adrain et al., 2012;McIlwain et al., 2012;Issuree et al., 2013)
TAX1BP1	B	1	+		+	(Shembade et al., 2007;Shembade et al., 2010;Shembade et al., 2011;Verstrepen et al., 2011;Nakano et al., 2013)
TNIP2	B	1	+			(Papoutsopoulou et al., 2006;Leotoing et al., 2011;Callahan et al., 2013)
ZFP36	B	1	+	+		(Liang et al., 2009;Schichl et al., 2009;Bros et al., 2010;Kang et al., 2011;Kaplan et al., 2011;Qian et al., 2011;Schott et al., 2014)
CD302	C	1				(Kato et al., 2007)
STUB1	D	8	+		+	(Kettern et al., 2011;Yang et al., 2011;Chen et al., 2013)
UCP2	D	8	+	+		(Bai et al., 2005;Haschemi et al., 2011)
SHARPIN	D	5	+		+	(Gerlach et al., 2011;Ikeda et al., 2011;Tokunaga et al., 2011;Sieber et al., 2012;Wang et al., 2012b;Pouwels et al., 2013)
RRAS	D	3	+			(Li et al., 2001;Shang et al., 2011;Singh et al., 2011)
RPS6KA3	D	2	+			(Lin et al., 2008;Kakugawa et al., 2009;Peng et al., 2010;Takada, 2015;Takada et al., 2016)
COMMD1	D	1	+		+	(Maine et al., 2007;Starokadomskyy et al., 2013;Bartuzi et al., 2014;O'Hara et al., 2014)
DPP3	D	1				(Gamrekelashvili et al., 2013)

Gene	Cluster	Freq.	NF-κB	IFN-γ	Ubiq.	References
ICAM2	D	1				(Somersalo et al., 1995;Helander et al., 1996;Lehmann et al., 2003;Banerjee et al., 2007;Porter and Hall, 2009;Boscacci et al., 2010)
MYBBP1A	D	1	+			(Owen et al., 2007;Cai et al., 2013)
PLIN2	D	1				(Bougnères et al., 2009)
RAD23A	D	1			+	(Andersson et al., 2005;Fang et al., 2013)
STK10	D	1	+			(Endo et al., 2000;Tao et al., 2002;Fukumura et al., 2013)

[a]Association suggested from Ingenuity Pathway Analysis in Figure 6

REFERENCES

Abhimanyu, Jha, P., Jain, A., Arora, K., and Bose, M. (2011). Genetic association study suggests a role for SP110 variants in lymph node tuberculosis but not pulmonary tuberculosis in north Indians. *Hum Immunol* 72**,** 576-580.

Adachi, O., Kawai, T., Takeda, K., Matsumoto, M., Tsutsui, H., Sakagami, M. et al. (1998). Targeted disruption of the *MyD88* gene results in loss of IL-1- and IL-18-mediated function. *Immunity* 9**,** 143-150.

Adrain, C., Zettl, M., Christova, Y., Taylor, N., and Freeman, M. (2012). Tumor necrosis factor signaling requires iRhom2 to promote trafficking and activation of TACE. *Science* 335**,** 225-228.

Andersson, H.A., Passeri, M.F., and Barry, M.A. (2005). Rad23 as a reciprocal agent for stimulating or repressing immune responses. *Hum Gene Ther* 16**,** 634-641.

Bai, Y., Onuma, H., Bai, X., Medvedev, A.V., Misukonis, M., Weinberg, J.B. et al. (2005). Persistent nuclear factor-kappa B activation in *Ucp2*-/- mice leads to enhanced nitric oxide and inflammatory cytokine production. *J Biol Chem* 280**,** 19062-19069.

Banerjee, P., Feuer, G., and Barker, E. (2007). Human T-cell leukemia virus type 1 (HTLV-1) p12I down-modulates ICAM-1 and -2 and reduces adherence of natural killer cells, thereby protecting HTLV-1-infected primary $CD4^+$ T cells from autologous natural killer cell-mediated cytotoxicity despite the reduction of major histocompatibility complex class I molecules on infected cells. *J Virol* 81**,** 9707-9717.

Bartuzi, P., Wijshake, T., Dekker, D.C., Fedoseienko, A., Kloosterhuis, N.J., Youssef, S.A. et al. (2014). A cell-type-specific role for murine Commd1 in liver inflammation. *Biochim. Biophys. Acta* 1842**,** 2257-2265.

Beattie, L., Phillips, R., Brown, N., Owens, B.M., Chauhan, N., Dalton, J.E. et al. (2011). Interferon regulatory factor 7 contributes to the control of *Leishmania donovani* in the mouse liver. *Infect. Immun* 79**,** 1057-1066.

Boehm, U., Klamp, T., Groot, M., and Howard, J.C. (1997). Cellular responses to interferon-γ. *Annu Rev Immunol* 15**,** 749-795.

Borg, C., Jalil, A., Laderach, D., Maruyama, K., Wakasugi, H., Charrier, S. et al. (2004). NK cell activation by dendritic cells (DCs) requires the formation of a synapse leading to IL-12 polarization in DCs. *Blood* 104**,** 3267-3275.

Boscacci, R.T., Pfeiffer, F., Gollmer, K., Sevilla, A.I., Martin, A.M., Soriano, S.F. et al. (2010). Comprehensive analysis of lymph node stroma-expressed Ig superfamily members reveals redundant and nonredundant roles for ICAM-1, ICAM-2, and VCAM-1 in lymphocyte homing. *Blood* 116**,** 915-925.

Boss, J.M. (1997). Regulation of transcription of MHC class II genes. *Curr Opin Immunol* 9**,** 107-113.

Bougnères, L., Helft, J., Tiwari, S., Vargas, P., Chang, B.H., Chan, L. et al. (2009). A role for lipid bodies in the cross-presentation of phagocytosed antigens by MHC class I in dendritic cells. *Immunity* 31**,** 232-244.

Bros, M., Wiechmann, N., Besche, V., Art, J., Pautz, A., Grabbe, S. et al. (2010). The RNA binding protein tristetraprolin influences the activation state of murine dendritic cells. *Mol Immunol* 47**,** 1161-1170.

Burns, K., Martinon, F., Esslinger, C., Pahl, H., Schneider, P., Bodmer, J.L. et al. (1998). MyD88, an adapter protein involved in interleukin-1 signaling. *J Biol Chem* 273**,** 12203-12209.

Cai, L., Deng, S.L., Liang, L., Pan, H., Zhou, J., Wang, M.Y. et al. (2013). Identification of genetic associations of *SP110/MYBBP1A/RELA* with pulmonary tuberculosis in the Chinese Han population. *Hum. Genet* 132**,** 265-273.

Callahan, J.A., Hammer, G.E., Agelides, A., Duong, B.H., Oshima, S., North, J. et al. (2013). Cutting edge: ABIN-1 protects against psoriasis by restricting MyD88 signals in dendritic cells. *J Immunol* 191**,** 535-539.

Carmody, R.J., Ruan, Q., Liou, H.C., and Chen, Y.H. (2007). Essential roles of c-Rel in TLR-induced IL-23 p19 gene expression in dendritic cells. *J Immunol* 178**,** 186-191.

Carracedo, A., Ito, K., and Pandolfi, P.P. (2011). The nuclear bodies inside out: PML conquers the cytoplasm. *Curr Opin Cell Biol* 23**,** 360-366.

Catucci, M., Zanoni, I., Draghici, E., Bosticardo, M., Castiello, M.C., Venturini, M. et al. (2014). Wiskott-Aldrich syndrome protein deficiency in natural killer and dendritic cells affects antitumor immunity. *Eur J Immunol* 44**,** 1039-1045.

Cenciarelli, C., Chiaur, D.S., Guardavaccaro, D., Parks, W., Vidal, M., and Pagano, M. (1999). Identification of a family of human F-box proteins. *Curr. Biol* 9**,** 1177-1179.

Chamberlain, J.W., Vasavada, H.A., Ganguly, S., and Weissman, S.M. (1991). Identification of cis sequences controlling efficient position-independent tissue-specific expression of human major histocompatibility complex class I genes in transgenic mice. *Mol Cell Biol* 11**,** 3564-3572.

Chau, T.L., Gioia, R., Gatot, J.S., Patrascu, F., Carpentier, I., Chapelle, J.P. et al. (2008). Are the IKKs and IKK-related kinases TBK1 and IKK-epsilon similarly activated? *Trends Biochem Sci* 33**,** 171-180.

Chen, A., Gao, B., Zhang, J., Mcewen, T., Ye, S.Q., Zhang, D. et al. (2009). The HECT-type E3 ubiquitin ligase AIP2 inhibits activation-induced T-cell death by catalyzing EGR2 ubiquitination. *Mol. Cell Biol* 29**,** 5348-5356.

Chen, Z., Barbi, J., Bu, S., Yang, H.Y., Li, Z., Gao, Y. et al. (2013). The ubiquitin ligase Stub1 negatively modulates regulatory T cell suppressive activity by promoting degradation of the transcription factor Foxp3. *Immunity* 39**,** 272-285.

Cohen, P. (2014). The TLR and IL-1 signalling network at a glance. *J Cell Sci* 127**,** 2383-2390.

Cohen, T.S., and Prince, A.S. (2013). Bacterial pathogens activate a common inflammatory pathway through IFNλ regulation of PDCD4. *PLoS. Pathog* 9**,** e1003682.

Crow, Y.J., Chase, D.S., Lowenstein, S.J., Szynkiewicz, M., Forte, G.M., Gornall, H.L. et al. (2015). Characterization of human disease phenotypes associated with mutations in *TREX1, RNASEH2A, RNASEH2B, RNASEH2C, SAMHD1, ADAR,* and *IFIH1*. *Am J Med Genet A* 167A**,** 296-312.

Endo, J., Toyama-Sorimachi, N., Taya, C., Kuramochi-Miyagawa, S., Nagata, K., Kuida, K. et al. (2000). Deficiency of a STE20/PAK family kinase LOK leads to the acceleration of LFA-1 clustering and cell adhesion of activated lymphocytes. *FEBS Lett* 468**,** 234-238.

Fang, D.F., He, K., Wang, J., Mu, R., Tan, B., Jian, Z. et al. (2013). RAD23A negatively regulates RIG-I/MDA5 signaling through promoting TRAF2 polyubiquitination and degradation. *Biochem. Biophys. Res. Commun* 431**,** 686-692.

Fox, G.J., Sy, D.N., Nhung, N.V., Yu, B., Ellis, M.K., N., V.H. et al. (2014). Polymorphisms of *SP110* are associated with both pulmonary and extra-pulmonary tuberculosis among the Vietnamese. *PLoS. One* 9**,** e99496.

Fukumura, K., Yamashita, Y., Kawazu, M., Sai, E., Fujiwara, S., Nakamura, N. et al. (2013). *STK10* missense mutations associated with anti-apoptotic function. *Oncol Rep* 30**,** 1542-1548.

Gamrekelashvili, J., Kapanadze, T., Han, M., Wissing, J., Ma, C., Jaensch, L. et al. (2013). Peptidases released by necrotic cells control $CD8^+$ T cell cross-priming. *J Clin Invest* 123**,** 4755-4768.

Gerlach, B., Cordier, S.M., Schmukle, A.C., Emmerich, C.H., Rieser, E., Haas, T.L. et al. (2011). Linear ubiquitination prevents inflammation and regulates immune signalling. *Nature* 471**,** 591-596.

Giacconi, R., Muti, E., Malavolta, M., Cipriano, C., Costarelli, L., Bernardini, G. et al. (2007). The +838 C/G *MT2A* polymorphism, metals, and the inflammatory/immune response in carotid artery stenosis in elderly people. *Mol. Med* 13**,** 388-395.

Girdlestone, J. (2000). Synergistic induction of HLA class I expression by RelA and CIITA. *Blood* 95**,** 3804-3808.

Girdlestone, J., Isamat, M., Gewert, D., and Milstein, C. (1993). Transcriptional regulation of *HLA-A* and *-B*: differential binding of members of the Rel and IRF families of transcription factors. *Proc Natl Acad Sci U S A* 90**,** 11568-11572.

Gismondi, A., Cifaldi, L., Mazza, C., Giliani, S., Parolini, S., Morrone, S. et al. (2004). Impaired natural and CD16-mediated NK cell cytotoxicity in patients with WAS and XLT: ability of IL-2 to correct NK cell functional defect. *Blood* 104**,** 436-443.

Gosling, K.M., Goodnow, C.C., Verma, N.K., and Fahrer, A.M. (2008). Defective T-cell function leading to reduced antibody production in a kleisin-beta mutant mouse. *Immunology* 125**,** 208-217.

Gosling, K.M., Makaroff, L.E., Theodoratos, A., Kim, Y.H., Whittle, B., Rui, L. et al. (2007). A mutation in a chromosome condensin II subunit, kleisin beta, specifically disrupts T cell development. *Proc Natl Acad Sci U S A* 104**,** 12445-12450.

Guo, L., Giasson, B.I., Glavis-Bloom, A., Brewer, M.D., Shorter, J., Gitler, A.D. et al. (2014). A cellular system that degrades misfolded proteins and protects against neurodegeneration. *Mol. Cell* 55**,** 15-30.

Gupta-Rossi, N., Storck, S., Griebel, P.J., Reynaud, C.A., Weill, J.C., and Dahan, A. (2003). Specific over-expression of deltex and a new Kelch-like protein in human germinal center B cells. *Mol Immunol* 39**,** 791-799.

Hakem, R., Le Bouteiller, P., Jezo-Bremond, A., Harper, K., Campese, D., and Lemonnier, F.A. (1991). Differential regulation of HLA-A3 and HLA-B7 MHC class I genes by IFN is due to two nucleotide differences in their IFN response sequences. *J Immunol* 147**,** 2384-2390.

Ham, H., Guerrier, S., Kim, J., Schoon, R.A., Anderson, E.L., Hamann, M.J. et al. (2013). Dedicator of cytokinesis 8 interacts with talin and Wiskott-Aldrich syndrome protein to regulate NK cell cytotoxicity. *J Immunol* 190**,** 3661-3669.

Hart, B.E., and Tapping, R.I. (2012). Cell surface trafficking of TLR1 is differentially regulated by the chaperones PRAT4A and PRAT4B. *J Biol. Chem* 287**,** 16550-16562.

Haschemi, A., Chin, B.Y., Jeitler, M., Esterbauer, H., Wagner, O., Bilban, M. et al. (2011). Carbon monoxide induced PPARγ SUMOylation and UCP2 block inflammatory gene expression in macrophages. *PLoS One* 6**,** e26376.

Helander, T.S., Carpen, O., Turunen, O., Kovanen, P.E., Vaheri, A., and Timonen, T. (1996). ICAM-2 redistributed by ezrin as a target for killer cells. *Nature* 382**,** 265-268.

Hilliard, A., Hilliard, B., Zheng, S.J., Sun, H., Miwa, T., Song, W. et al. (2006). Translational regulation of autoimmune inflammation and lymphoma genesis by programmed cell death 4. *J Immunol* 177**,** 8095-8102.

Huang, W., Ochs, H.D., Dupont, B., and Vyas, Y.M. (2005). The Wiskott-Aldrich syndrome protein regulates nuclear translocation of NFAT2 and NF-κB (RelA) independently of its role in filamentous actin polymerization and actin cytoskeletal rearrangement. *J Immunol* 174**,** 2602-2611.

Huang, Y.C., Li, Z., Carter, J.D., Soukup, J.M., Schwartz, D.A., and Yang, I.V. (2009). Fine ambient particles induce oxidative stress and metal binding genes in human alveolar macrophages. *Am J Respir Cell Mol Biol* 41**,** 544-552.

Ikeda, F., Deribe, Y.L., Skanland, S.S., Stieglitz, B., Grabbe, C., Franz-Wachtel, M. et al. (2011). SHARPIN forms a linear ubiquitin ligase complex regulating NF-κB activity and apoptosis. *Nature* 471**,** 637-641.

Issuree, P.D., Maretzky, T., Mcilwain, D.R., Monette, S., Qing, X., Lang, P.A. et al. (2013). iRHOM2 is a critical pathogenic mediator of inflammatory arthritis. *J Clin Invest* 123**,** 928-932.

Jin, P., Han, T.H., Ren, J., Saunders, S., Wang, E., Marincola, F.M. et al. (2010). Molecular signatures of maturing dendritic cells: implications for testing the quality of dendritic cell therapies. *J Transl Med* 8**,** 4.

Johnson, D.R. (2003). Locus-specific constitutive and cytokine-induced HLA class I gene expression. *J Immunol* 170**,** 1894-1902.

Kakugawa, S., Shimojima, M., Goto, H., Horimoto, T., Oshimori, N., Neumann, G. et al. (2009). Mitogen-activated protein kinase-activated kinase RSK2 plays a role in innate immune responses to influenza virus infection. *J Virol* 83**,** 2510-2517.

Kang, J.G., Amar, M.J., Remaley, A.T., Kwon, J., Blackshear, P.J., Wang, P.Y. et al. (2011). Zinc finger protein tristetraprolin interacts with CCL3 mRNA and regulates tissue inflammation. *J Immunol* 187**,** 2696-2701.

Kaplan, I.M., Morisot, S., Heiser, D., Cheng, W.C., Kim, M.J., and Civin, C.I. (2011). Deletion of tristetraprolin caused spontaneous reactive granulopoiesis by a non-cell-autonomous mechanism without disturbing long-term hematopoietic stem cell quiescence. *J Immunol* 186**,** 2826-2834.

Kato, M., Khan, S., D'aniello, E., Mcdonald, K.J., and Hart, D.N. (2007). The novel endocytic and phagocytic C-Type lectin receptor DCL-1/CD302 on macrophages is colocalized with F-actin, suggesting a role in cell adhesion and migration. *J Immunol* 179**,** 6052-6063.

Kawai, T., Sato, S., Ishii, K.J., Coban, C., Hemmi, H., Yamamoto, M. et al. (2004). Interferon-α induction through Toll-like receptors involves a direct interaction of IRF7 with MyD88 and TRAF6. *Nat Immunol* 5**,** 1061-1068.

Kettern, N., Rogon, C., Limmer, A., Schild, H., and Hohfeld, J. (2011). The Hsc/Hsp70 co-chaperone network controls antigen aggregation and presentation during maturation of professional antigen presenting cells. *PLoS One* 6**,** e16398.

Konno, K., Wakabayashi, Y., Akashi-Takamura, S., Ishii, T., Kobayashi, M., Takahashi, K. et al. (2006). A molecule that is associated with Toll-like receptor 4 and regulates its cell surface expression. *Biochem. Biophys. Res. Commun* 339**,** 1076-1082.

Kroll, J., Shi, X., Caprioli, A., Liu, H.H., Waskow, C., Lin, K.M. et al. (2005). The BTB-kelch protein KLHL6 is involved in B-lymphocyte antigen receptor signaling and germinal center formation. *Mol Cell Biol* 25**,** 8531-8540.

Krzewski, K., Chen, X., Orange, J.S., and Strominger, J.L. (2006). Formation of a WIP-, WASp-, actin-, and myosin IIA-containing multiprotein complex in activated NK cells and its alteration by KIR inhibitory signaling. *J Cell Biol* 173**,** 121-132.

Lee, S.M., Kim, E.J., Suk, K., and Lee, W.H. (2011). CD300F blocks both MyD88 and TRIF-mediated TLR signaling through activation of Src homology region 2 domain-containing phosphatase 1. *J Immunol* 186**,** 6296-6303.

Lehmann, J.C., Jablonski-Westrich, D., Haubold, U., Gutierrez-Ramos, J.C., Springer, T., and Hamann, A. (2003). Overlapping and selective roles of endothelial intercellular adhesion molecule-1 (ICAM-1) and ICAM-2 in lymphocyte trafficking. *J Immunol* 171**,** 2588-2593.

Leotoing, L., Chereau, F., Baron, S., Hube, F., Valencia, H.J., Bordereaux, D. et al. (2011). A20-binding inhibitor of nuclear factor-κB (NF-κB)-2 (ABIN-2) is an activator of inhibitor of NF-κB (IκB) kinase α (IKKα)-mediated NF-κB transcriptional activity. *J Biol Chem* 286**,** 32277-32288.

Li, J., Peet, G.W., Balzarano, D., Li, X., Massa, P., Barton, R.W. et al. (2001). Novel NEMO/IκB kinase and NF-κB target genes at the pre-B to immature B cell transition. *J Biol Chem* 276**,** 18579-18590.

Liang, J., Lei, T., Song, Y., Yanes, N., Qi, Y., and Fu, M. (2009). RNA-destabilizing factor tristetraprolin negatively regulates NF-κB signaling. *J Biol Chem* 284**,** 29383-29390.

Lin, J.X., Spolski, R., and Leonard, W.J. (2008). Critical role for Rsk2 in T-lymphocyte activation. *Blood* 111**,** 525-533.

Liu, B., Yang, Y., Qiu, Z., Staron, M., Hong, F., Li, Y. et al. (2010). Folding of Toll-like receptors by the HSP90 paralogue gp96 requires a substrate-specific cochaperone. *Nat Commun* 1**,** 79.

Liu, L., Tran, E., Zhao, Y., Huang, Y., Flavell, R., and Lu, B. (2005). Gadd45β and Gadd45γ are critical for regulating autoimmunity. *J Exp. Med* 202**,** 1341-1347.

Maine, G.N., Mao, X., Komarck, C.M., and Burstein, E. (2007). COMMD1 promotes the ubiquitination of NF-κB subunits through a cullin-containing ubiquitin ligase. *EMBO J* 26**,** 436-447.

Mauro, C., Leow, S.C., Anso, E., Rocha, S., Thotakura, A.K., Tornatore, L. et al. (2011). NF-κB controls energy homeostasis and metabolic adaptation by upregulating mitochondrial respiration. *Nat Cell Biol* 13**,** 1272-1279.

Mcilwain, D.R., Lang, P.A., Maretzky, T., Hamada, K., Ohishi, K., Maney, S.K. et al. (2012). iRhom2 regulation of TACE controls TNF-mediated protection against Listeria and responses to LPS. *Science* 335**,** 229-232.

Min, W., Pober, J.S., and Johnson, D.R. (1996). Kinetically coordinated induction of TAP1 and HLA class I by IFN-γ: the rapid induction of TAP1 by IFN-γ is mediated by Stat1α. *J Immunol* 156**,** 3174-3183.

Nakano, S., Ikebe, E., Tsukamoto, Y., Wang, Y., Matsumoto, T., Mitsui, T. et al. (2013). Commensal microbiota contributes to chronic endocarditis in TAX1BP1 deficient mice. *PLoS. One* 8**,** e73205.

Norman, P.J., Hollenbach, J.A., Nemat-Gorgani, N., Guethlein, L.A., Hilton, H.G., Pando, M.J. et al. (2013). Co-evolution of human leukocyte antigen (HLA) class I ligands with killer-cell immunoglobulin-like receptors (KIR) in a genetically diverse population of sub-Saharan Africans. *PLoS. Genet* 9**,** e1003938.

O'hara, A., Simpson, J., Morin, P., Loveridge, C.J., Williams, A.C., Novo, S.M. et al. (2014). p300-mediated acetylation of COMMD1 regulates its stability, and the ubiquitylation and nucleolar translocation of the RelA NF-κB subunit. *J Cell Sci* 127**,** 3659-3665.

Ogawa, S., Lozach, J., Benner, C., Pascual, G., Tangirala, R.K., Westin, S. et al. (2005). Molecular determinants of crosstalk between nuclear receptors and toll-like receptors. *Cell* 122**,** 707-721.

Orange, J.S., Ramesh, N., Remold-O'donnell, E., Sasahara, Y., Koopman, L., Byrne, M. et al. (2002). Wiskott-Aldrich syndrome protein is required for NK cell cytotoxicity and colocalizes with actin to NK cell-activating immunologic synapses. *Proc. Natl. Acad. Sci. U. S. A* 99**,** 11351-11356.

Orr, M.T., and Lanier, L.L. (2011). Natural killer cell licensing during viral infection. *Adv. Exp. Med. Biol* 780**,** 37-44.

Orr, M.T., Murphy, W.J., and Lanier, L.L. (2010). 'Unlicensed' natural killer cells dominate the response to cytomegalovirus infection. *Nat. Immunol* 11**,** 321-327.

Othman, S., Rahman, N.A., and Yusof, R. (2012). Induction of MHC Class I HLA-A2 promoter by dengue virus occurs at the NF-κB binding domains of the Class I Regulatory Complex. *Virus Res* 163**,** 238-245.

Owen, H.R., Elser, M., Cheung, E., Gersbach, M., Kraus, W.L., and Hottiger, M.O. (2007). MYBBP1a is a novel repressor of NF-κB. *J Mol. Biol* 366**,** 725-736.

Pan, H., Yan, B.S., Rojas, M., Shebzukhov, Y.V., Zhou, H., Kobzik, L. et al. (2005). *Ipr1* gene mediates innate immunity to tuberculosis. *Nature* 434**,** 767-772.

Pan, Y., Huang, J., Xing, R., Yin, X., Cui, J., Li, W. et al. (2013). Metallothionein 2A inhibits NF-κB pathway activation and predicts clinical outcome segregated with TNM stage in gastric cancer patients following radical resection. *J Transl. Med* 11**,** 173.

Papa, S., Zazzeroni, F., Bubici, C., Jayawardena, S., Alvarez, K., Matsuda, S. et al. (2004). Gadd45β mediates the NF-κB suppression of JNK signalling by targeting MKK7/JNKK2. *Nat Cell Biol* 6**,** 146-153.

Papoutsopoulou, S., Symons, A., Tharmalingham, T., Belich, M.P., Kaiser, F., Kioussis, D. et al. (2006). ABIN-2 is required for optimal activation of Erk MAP kinase in innate immune responses. *Nat Immunol* 7**,** 606-615.

Peng, C., Cho, Y.Y., Zhu, F., Xu, Y.M., Wen, W., Ma, W.Y. et al. (2010). RSK2 mediates NF-κB activity through the phosphorylation of IκBα in the TNF-R1 pathway. *FASEB J* 24**,** 3490-3499.

Porter, J.C., and Hall, A. (2009). Epithelial ICAM-1 and ICAM-2 regulate the egression of human T cells across the bronchial epithelium. *FASEB J* 23**,** 492-502.

Pouwels, J., De Franceschi, N., Rantakari, P., Auvinen, K., Karikoski, M., Mattila, E. et al. (2013). SHARPIN regulates uropod detachment in migrating lymphocytes. *Cell Rep* 5**,** 619-628.

Qian, X., Ning, H., Zhang, J., Hoft, D.F., Stumpo, D.J., Blackshear, P.J. et al. (2011). Posttranscriptional regulation of IL-23 expression by IFN-γ through tristetraprolin. *J Immunol* 186**,** 6454-6464.

Randow, F., and Seed, B. (2001). Endoplasmic reticulum chaperone gp96 is required for innate immunity but not cell viability. *Nat Cell Biol* 3**,** 891-896.

Rice, G.I., Forte, G.M., Szynkiewicz, M., Chase, D.S., Aeby, A., Abdel-Hamid, M.S. et al. (2013). Assessment of interferon-related biomarkers in Aicardi-Goutières syndrome associated with mutations in *TREX1*, *RNASEH2A*, *RNASEH2B*, *RNASEH2C*, *SAMHD1*, and *ADAR*: a case-control study. *Lancet Neurol* 12**,** 1159-1169.

Sarkar, K., Sadhukhan, S., Han, S.S., and Vyas, Y.M. (2014). Disruption of hSWI/SNF complexes in T cells by WAS mutations distinguishes X-linked thrombocytopenia from Wiskott-Aldrich syndrome. *Blood* 124**,** 3409-3419.

Sarkar, K., Sadhukhan, S., Han, S.S., and Vyas, Y.M. (2015). SUMOylation-disrupting WAS mutation converts WASp from a transcriptional activator to a repressor of NF-κB response genes in T cells. *Blood* 126**,** 1670-1682.

Schaeffer, V., Akutsu, M., Olma, M.H., Gomes, L.C., Kawasaki, M., and Dikic, I. (2014). Binding of OTULIN to the PUB domain of HOIP controls NF-κB signaling. *Mol. Cell* 54**,** 349-361.

Schichl, Y.M., Resch, U., Hofer-Warbinek, R., and De Martin, R. (2009). Tristetraprolin impairs NF-κB/p65 nuclear translocation. *J Biol Chem* 284**,** 29571-29581.

Schott, J., Reitter, S., Philipp, J., Haneke, K., Schafer, H., and Stoecklin, G. (2014). Translational regulation of specific mRNAs controls feedback inhibition and survival during macrophage activation. *PLoS. Genet* 10**,** e1004368.

Serrano-Pertierra, E., Cernuda-Morollon, E., and Lopez-Larrea, C. (2012). Wiskott-Aldrich syndrome protein (WASp) and N-WASp are involved in the regulation of NK-cell migration upon NKG2D activation. *Eur J Immunol* 42**,** 2142-2151.

Shang, X., Cancelas, J.A., Li, L., Guo, F., Liu, W., Johnson, J.F. et al. (2011). R-Ras and Rac GTPase cross-talk regulates hematopoietic progenitor cell migration, homing, and mobilization. *J Biol Chem* 286**,** 24068-24078.

Sheedy, F.J., Palsson-Mcdermott, E., Hennessy, E.J., Martin, C., O'leary, J.J., Ruan, Q. et al. (2010). Negative regulation of TLR4 via targeting of the proinflammatory tumor suppressor PDCD4 by the microRNA miR-21. *Nat Immunol* 11**,** 141-147.

Sheikh, S.Z., Kobayashi, T., Matsuoka, K., Onyiah, J.C., and Plevy, S.E. (2011). Characterization of an interferon-stimulated response element (ISRE) in the *Il23α* promoter. *J Biol Chem* 286**,** 1174-1180.

Shembade, N., Harhaj, N.S., Liebl, D.J., and Harhaj, E.W. (2007). Essential role for TAX1BP1 in the termination of TNF-α-, IL-1- and LPS-mediated NF-κB and JNK signaling. *EMBO J* 26**,** 3910-3922.

Shembade, N., Ma, A., and Harhaj, E.W. (2010). Inhibition of NF-κB signaling by A20 through disruption of ubiquitin enzyme complexes. *Science* 327**,** 1135-1139.

Shembade, N., Pujari, R., Harhaj, N.S., Abbott, D.W., and Harhaj, E.W. (2011). The kinase IKKα inhibits activation of the transcription factor NF-κB by phosphorylating the regulatory molecule TAX1BP1. *Nat Immunol* 12**,** 834-843.

Shen, Y., Xia, M., Zhang, J., Xu, L., Yang, J., Chen, A. et al. (2009). IRF-1 and p65 mediate upregulation of constitutive HLA-A antigen expression by hepatocellular carcinoma cells. *Mol Immunol* 46**,** 2045-2053.

Sieber, S., Lange, N., Kollmorgen, G., Erhardt, A., Quaas, A., Gontarewicz, A. et al. (2012). Sharpin Contributes to TNF-α Dependent NF-κB Activation and Anti-Apoptotic Signalling in Hepatocytes. *PLoS One* 7**,** e29993.

Singh, G., Hashimoto, D., Yan, X., Helft, J., Park, P.J., Ma, G. et al. (2011). R-Ras is required for murine dendritic cell maturation and $CD4^+$ T-cell priming. *Blood* 10.1182/blood-2011-05-357319.

Sleiman, M., Brons, N.H., Kaoma, T., Dogu, F., Villa-Forte, A., Lenoble, P. et al. (2014). NK cell killer Ig-like receptor repertoire acquisition and maturation are strongly modulated by HLA class I molecules. *J Immunol* 192**,** 2602-2610.

Somersalo, K., Carpen, O., Saksela, E., Gahmberg, C.G., Nortamo, P., and Timonen, T. (1995). Activation of natural killer cell migration by leukocyte integrin-binding peptide from intracellular adhesion molecule-2 (ICAM-2). *J Biol. Chem* 270**,** 8629-8636.

Starokadomskyy, P., Gluck, N., Li, H., Chen, B., Wallis, M., Maine, G.N. et al. (2013). CCDC22 deficiency in humans blunts activation of proinflammatory NF-κB signaling. *J Clin Invest* 123**,** 2244-2256.

Staron, M., Yang, Y., Liu, B., Li, J., Shen, Y., Zuniga-Pflucker, J.C. et al. (2010). gp96, an endoplasmic reticulum master chaperone for integrins and Toll-like receptors, selectively regulates early T and B lymphopoiesis. *Blood* 115**,** 2380-2390.

Steimle, V., Siegrist, C.A., Mottet, A., Lisowska-Grospierre, B., and Mach, B. (1994). Regulation of MHC class II expression by interferon-gamma mediated by the transactivator gene CIITA. *Science* 265, 106-109.

Stieglitz, B., Rana, R.R., Koliopoulos, M.G., Morris-Davies, A.C., Schaeffer, V., Christodoulou, E. et al. (2013). Structural basis for ligase-specific conjugation of linear ubiquitin chains by HOIP. *Nature* 503, 422-426.

Takada, I. (2015). DGCR14 induces *Il17a* gene expression through the RORγ/BAZ1B/RSKS2 complex. *Mol Cell Biol* 35, 344-355.

Takada, I., Yogiashi, Y., and Makishima, M. (2016). The ribosomal S6 kinase inhibitor BI-D1870 ameliorated experimental autoimmune encephalomyelitis in mice. *Immunobiology* 221, 188-192.

Takekawa, M., and Saito, H. (1998). A family of stress-inducible GADD45-like proteins mediate activation of the stress-responsive MTK1/MEKK4 MAPKKK. *Cell* 95, 521-530.

Tao, L., Wadsworth, S., Mercer, J., Mueller, C., Lynn, K., Siekierka, J. et al. (2002). Opposing roles of serine/threonine kinases MEKK1 and LOK in regulating the CD28 responsive element in T-cells. *Biochem J* 363, 175-182.

Teng, M.W., Andrews, D.M., Mclaughlin, N., Von, S.B., Ngiow, S.F., Moller, A. et al. (2010). IL-23 suppresses innate immune response independently of IL-17A during carcinogenesis and metastasis. *Proc. Natl. Acad. Sci. U. S. A* 107, 8328-8333.

Thyss, R., Virolle, V., Imbert, V., Peyron, J.F., Aberdam, D., and Virolle, T. (2005). NF-κB/Egr-1/Gadd45 are sequentially activated upon UVB irradiation to mediate epidermal cell death. *EMBO J* 24, 128-137.

Toh, P.P., Li, J.J., Yip, G.W., Lo, S.L., Guo, C.H., Phan, T.T. et al. (2010). Modulation of metallothionein isoforms is associated with collagen deposition in proliferating keloid fibroblasts in vitro. *Exp Dermatol* 19, 987-993.

Tokunaga, F., Nakagawa, T., Nakahara, M., Saeki, Y., Taniguchi, M., Sakata, S. et al. (2011). SHARPIN is a component of the NF-κB-activating linear ubiquitin chain assembly complex. *Nature* 471, 633-636.

Tosh, K., Campbell, S.J., Fielding, K., Sillah, J., Bah, B., Gustafson, P. et al. (2006). Variants in the *SP110* gene are associated with genetic susceptibility to tuberculosis in West Africa. *Proc. Natl. Acad. Sci. U. S. A* 103, 10364-10368.

Tsui, F.W., Martin, A., Wang, J., and Tsui, H.W. (2006). Investigations into the regulation and function of the SH2 domain-containing protein-tyrosine phosphatase, SHP-1. *Immunol Res* 35, 127-136.

Tsukahara, F., and Maru, Y. (2010). Bag1 directly routes immature BCR-ABL for proteasomal degradation. *Blood* 116**,** 3582-3592.

Vandeghinste, N., Proost, P., and De Ley, M. (2000). Metallothionein isoform gene expression in zinc-treated human peripheral blood lymphocytes. *Cell Mol Biol (Noisy-le-grand)* 46**,** 419-433.

Verstrepen, L., Verhelst, K., Carpentier, I., and Beyaert, R. (2011). TAX1BP1, a ubiquitin-binding adaptor protein in innate immunity and beyond. *Trends Biochem Sci* 36**,** 347-354.

Wang, Y., Zhu, Z., Church, T.D., Lugogo, N.L., Que, L.G., Francisco, D. et al. (2012a). SHP-1 as a critical regulator of *Mycoplasma pneumoniae*-induced inflammation in human asthmatic airway epithelial cells. *J Immunol* 188**,** 3371-3381.

Wang, Z., Sokolovska, A., Seymour, R., Sundberg, J.P., and Hogenesch, H. (2012b). SHARPIN is essential for cytokine production, NF-κB signaling, and induction of Th1 differentiation by dendritic cells. *PLoS. One* 7**,** e31809.

Westerheide, S.D., Louis-Plence, P., Ping, D., He, X.F., and Boss, J.M. (1997). *HLA-DMA* and *HLA-DMB* gene expression functions through the conserved S-X-Y region. *J Immunol* 158**,** 4812-4821.

Winston, J.T., Koepp, D.M., Zhu, C., Elledge, S.J., and Harper, J.W. (1999). A family of mammalian F-box proteins. *Curr. Biol* 9**,** 1180-1182.

Wu, W.S., Xu, Z.X., Hittelman, W.N., Salomoni, P., Pandolfi, P.P., and Chang, K.S. (2003). Promyelocytic leukemia protein sensitizes tumor necrosis factor α-induced apoptosis by inhibiting the NF-κB survival pathway. *J Biol Chem* 278**,** 12294-12304.

Yamanaka, Y., Tagawa, H., Takahashi, N., Watanabe, A., Guo, Y.M., Iwamoto, K. et al. (2009). Aberrant overexpression of microRNAs activate AKT signaling via down-regulation of tumor suppressors in natural killer-cell lymphoma/leukemia. *Blood* 114**,** 3265-3275.

Yang, I.V., Wade, C.M., Kang, H.M., Alper, S., Rutledge, H., Lackford, B. et al. (2009). Identification of novel genes that mediate innate immunity using inbred mice. *Genetics* 183**,** 1535-1544.

Yang, M., Wang, C., Zhu, X., Tang, S., Shi, L., Cao, X. et al. (2011). E3 ubiquitin ligase CHIP facilitates Toll-like receptor signaling by recruiting and polyubiquitinating Src and atypical PKCζ. *J Exp Med* 208**,** 2099-2112.

Yang, Y., Liao, B., Wang, S., Yan, B., Jin, Y., Shu, H.B. et al. (2013). E3 ligase WWP2 negatively regulates TLR3-mediated innate immune response by targeting TRIF for ubiquitination and degradation. *Proc. Natl. Acad. Sci. U. S. A* 110**,** 5115-5120.

Yang, Y., Liu, B., Dai, J., Srivastava, P.K., Zammit, D.J., Lefrancois, L. et al. (2007). Heat shock protein gp96 is a master chaperone for toll-like receptors and is important in the innate function of macrophages. *Immunity* 26**,** 215-226.

4 SUPPLEMENTARY TABLE 3

Genes/probe sets used in the IFN-driven modeling

Gene	Probe set ID	Frequency of use in models	Cluster
BAK1	203728_at	14	H
BAX[a,b]	208478_s_at	14	H
BAX[a]	211833_s_at	2	H
BCL2[a,b]	207004_at	52	G
BCL2[a]	203685_at	8	E
BCL2[a]	203684_s_at	1	E
CCL8	214038_at	11	E
CXCL10	204533_at	10	H
DDX58[a,b]	242961_x_at	24	E
DDX58[a]	218943_s_at	13	F
DDX58[a]	222793_at	5	H
EIF2AK2[a,b]	213294_at	3	H
EIF2AK2[a]	204211_x_at	2	H
GBP1[a,b]	202269_x_at	20	H
GBP1[a]	231577_s_at	9	H
GBP1[a]	202270_at	5	H
IFI16[a,b]	208965_s_at	12	F
IFI16[a]	206332_s_at	2	H
IFI16[a]	208966_x_at	2	H
IFI35	209417_s_at	3	H
IFI44[a,b]	214059_at	7	H
IFI44[a,c]	214453_s_at	0	
IFIH1[a,b]	219209_at	15	H
IFIH1[a]	1555464_at	2	H
IFIT1[c]	203153_at	0	
IFIT2	226757_at	6	H
IFIT3[a,b]	204747_at	4	H
IFIT3[a]	229450_at	2	H
IFITM1[a,b]	201601_x_at	1	F
IFITM1[a,c]	214022_s_at	0	
IFITM2	201315_x_at	3	F
IFITM3	212203_x_at	1	F
IFNGR1[a,b]	242903_at	6	E
IFNGR1[a]	202727_s_at	2	E
IFNGR1[a,c]	211676_s_at	0	
IFNGR2	201642_at	2	H

Gene	Probe set ID	Frequency of use in models	Cluster
IRF1[a,b]	238725_at	27	F
IRF1[a]	202531_at	13	F
IRF7	208436_s_at	36	F
IRF9	203882_at	9	F
ISG15	205483_s_at	30	H
ISG20[a,b]	33304_at	1	F
ISG20[a,c]	204698_at	0	
JAK1[a,b]	240613_at	67	G
JAK1[a]	201648_at	45	G
JAK1[a]	1552611_a_at	44	G
JAK1[a]	239695_at	13	E
JAK1[a]	1552610_a_at	3	G
JAK2[a,b]	205842_s_at	16	H
JAK2[a]	205841_at	11	H
JAK2[a]	1562031_at	5	H
MT1E	212859_x_at	10	F
MT1G	204745_x_at	7	F
MT2A	212185_x_at	66	F
MX1	202086_at	13	F
MX2	204994_at	2	H
OAS1[a,b]	205552_s_at	8	H
OAS1[a,c]	202869_at	0	
OAS2[a,b]	228607_at	12	F
OAS2[a]	204972_at	2	H
OAS2[a]	206553_at	1	H
OAS3	218400_at	3	H
OASL[a,b]	210797_s_at	12	H
OASL[a]	205660_at	2	H
PIAS1[a,b]	217864_s_at	24	G
PIAS1[a]	217862_at	6	E
PIAS1[a]	217863_at	1	E
PML	235508_at	8	F
PSMB8	209040_s_at	11	H
PTPN2[a,b]	241623_at	11	E
PTPN2[a]	241622_at	5	E
PTPN2[a]	241983_at	5	E
PTPN2[a,c]	213136_at	0	
PTPN2[a,c]	213137_s_at	0	
RELA[a,b]	201783_s_at	42	H
RELA[a]	230202_at	5	H

Gene	Probe set ID	Frequency of use in models	Cluster
RTP4	219684_at	15	H
SOCS1[a,b]	210001_s_at	34	H
SOCS1[a]	213337_s_at	8	H
STAT1[a,b]	200887_s_at	44	F
STAT1[a]	209969_s_at	15	F
TAP1	202307_s_at	79	F

[a]For genes with more than one probe set, the data from the [b]probe set that was most frequently represented in the models were considered as the representative data for that gene in the manuscript.
[c]Genes/probe sets that were not selected by the modeling process.

5 SUPPLEMENTARY FIGURE 1

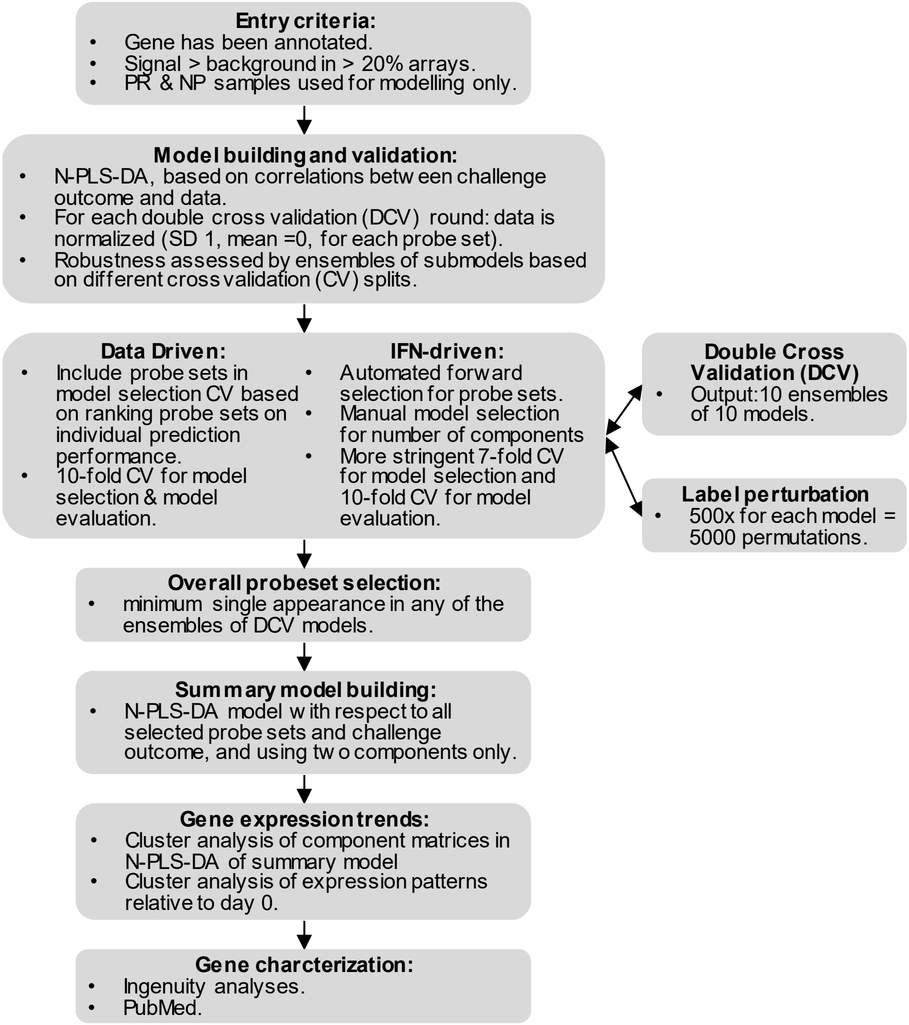

Flow diagram describing how the N-PLS-DA was conducted.

6 SUPPLEMENTARY FIGURE 2

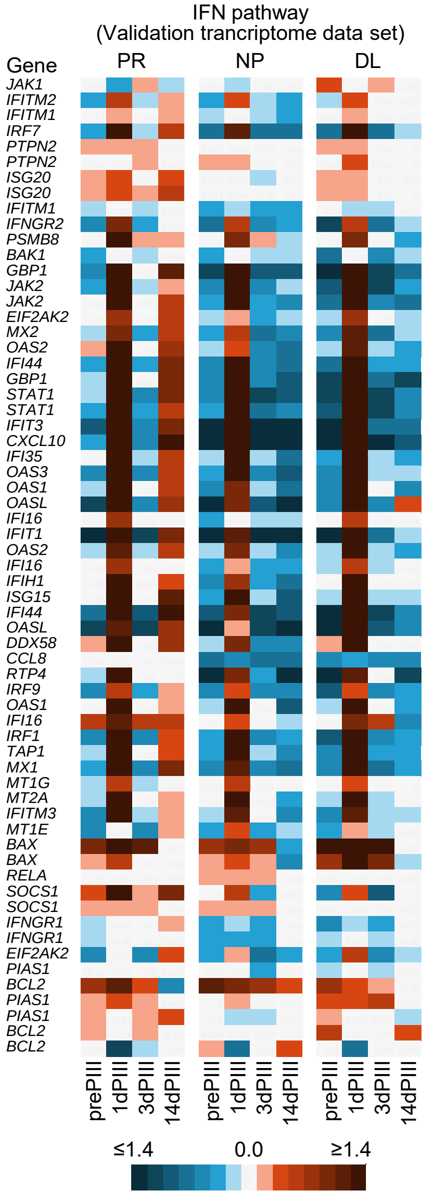

The evaluation of IFN-pathway gene expression for a potential microarray-batch effect using the validation-transcriptome data set.

Unlike the principal transcriptome data set, the validation transcriptome data set was generated from a single kit of microarrays. The heatmap describes IFN pathway gene expression in protected (PR), non-protected (NP) and non-protected with delayed onset of parasitemia (DL) groups before (prePIII) and 1, 3 and 14 days after the third vaccine injection (1dPIII, 3dPIII and 14dPIII, respectively). Mean RNA expression relative to prePI is described in accordance with the colored scale. Certain genes are represented by more than one probe set.

7 SUPPLEMENTARY FIGURE 3

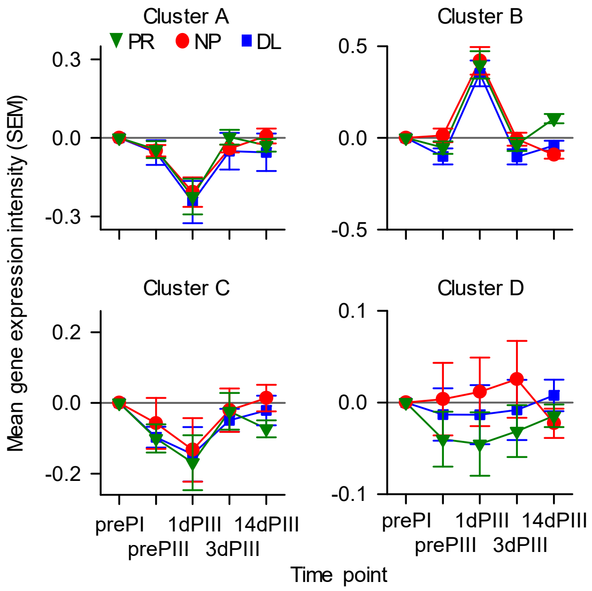

Evaluation of the expression of Clusters A to D probe sets for a potential microarray-batch effect using the validation transcriptome data set.

Unlike the principal transcriptome data set, the validation transcriptome data set was generated from a single kit of microarrays (Vahey et al., 2010). Mean RNA-expression levels relative to pre-dose 1 (prePI), at pre-dose 3 [prePIII] and 1, 3 and 14 days after dose 3 [1dPIII, 3dPIII, and 14d PIII, respectively])., with respect to protection status of subjects (protected [PR], non-protected [NP] and non-protected with delayed parasitemia [DL]) for each of the four clusters (A–D) of probe sets among the 116 probe sets (110 genes) identified by the data-driven model. The error bars indicate the standard error of the mean (SEM). Also, simulated modeling suggested that such a batch effect (which may have confounded the effect of identifying protection status at 14dPIII) would have been mitigated by the N-PLS-DA because data from several time points were included (not shown).
